# Supplementary material for: Widespread position-dependent transcriptional regulatory sequences in plants
Source: Nat Genet. 2024 Sep 12;56(10):2238–46. doi: 10.1038/s41588-024-01907-3 (PMC11525189; doi:10.1038/s41588-024-01907-3)
Supplement: Supplementary file 1 — Supplementary Figs. 1–22 and Note 1. [file 41588_2024_1907_MOESM1_ESM.pdf]

nature genetics

Supplementary information https://doi.org/10.1038/s41588-024-01907-3

Widespread position-dependent transcriptional regulatory sequences in plants

In the format provided by the authors and unedited

Supplementary Materials:

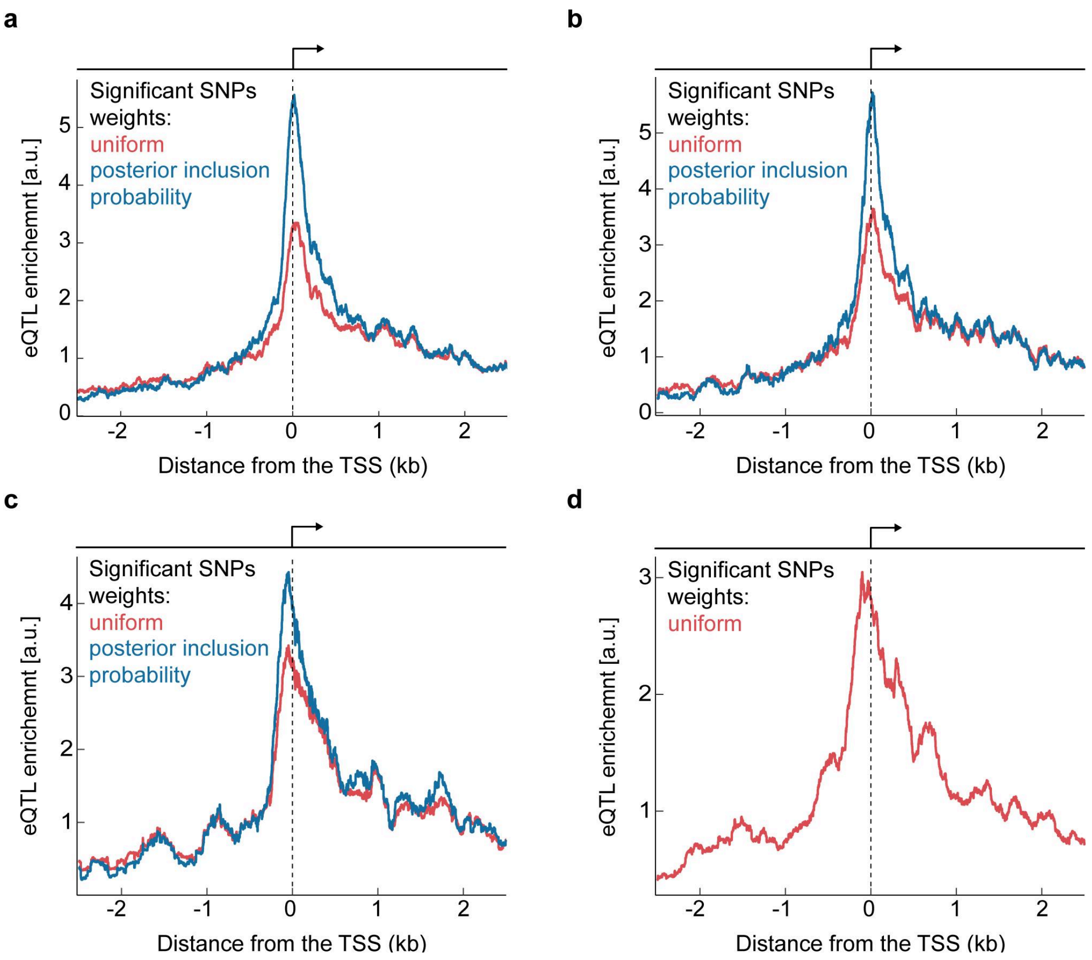

Supplementary Figure 1: Consistent eQTL enrichment near the TSS of genes across multiple datasets

eQTL enrichment near the transcription start site (TSS) of genes from various data sets: **(a)** Second batch from ref. [1] (4,259 genes with significant SNPs), **(b)** First batch from ref. [1] (2,760 genes with significant SNPs) **(c)** Ref. [2] (639 genes with significant SNPs), and **(d)** Ref. [3] (3,048 genes with significant SNPs). SNPs linked to the expression of the same gene were normalized to ensure equal contribution from each gene in the analysis. Weights were assigned either uniformly (red line), or based on the posterior inclusion probability (PIP, blue line), which accounts for linkage disequilibrium (LD) between SNPs. Incorporating LD using PIP consistently enhanced the enrichment in and downstream of the TSS across datasets. Data smoothed with a 100 bp (data from ref. [1]) or 200 bp (otherwise) rolling window.

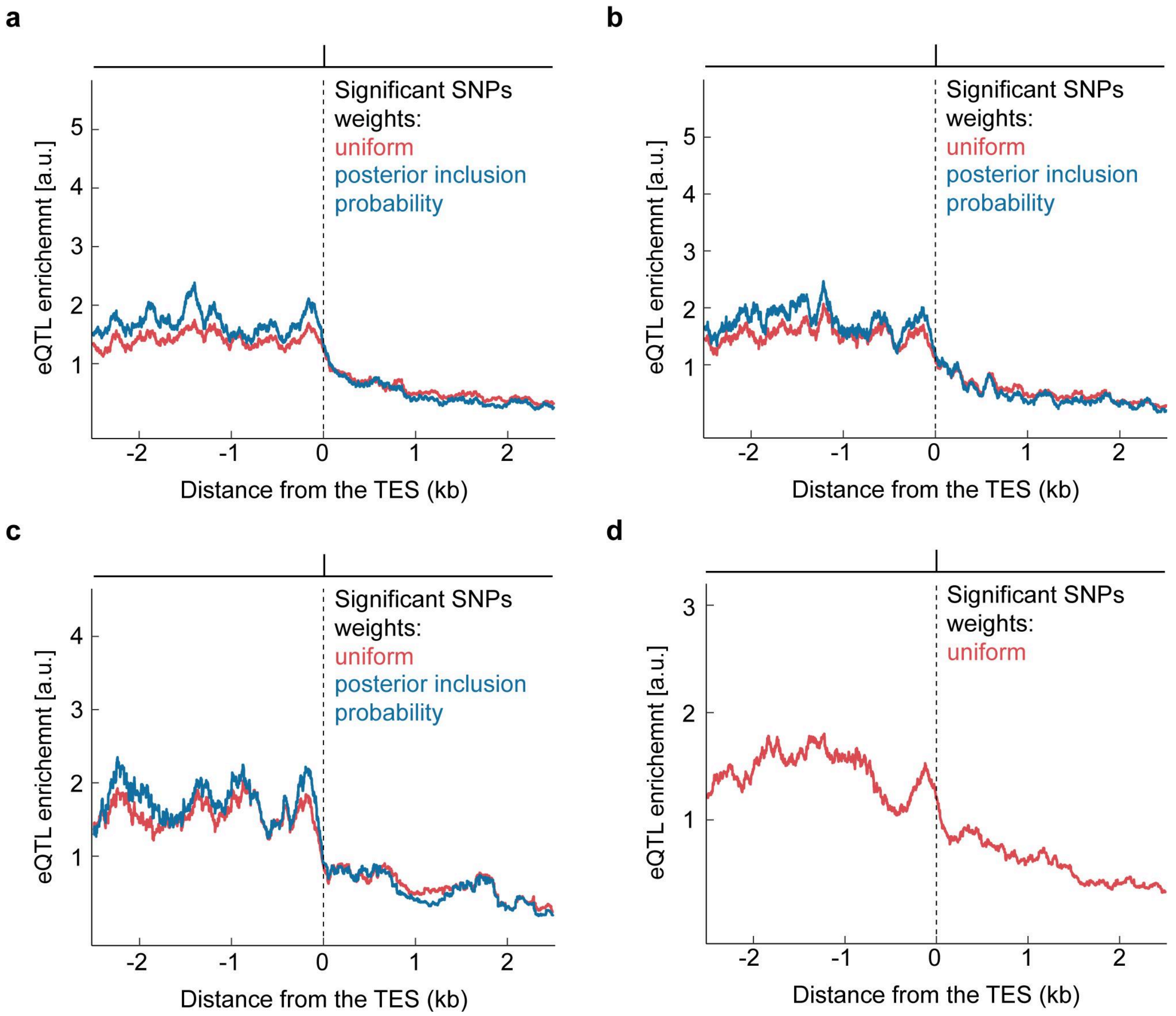

Supplementary Figure 2: eQTL enrichment near the transcription end site of genes

eQTL enrichment is depicted near the transcript 3' end site (TES) of genes from various datasets, using the same methodology and details as described in Supplementary Fig. 1. The y-axis scaling is the same as in the respective sub-plot in Supplementary Fig. 1 to enable comparisons between the figures. The enrichment observed near the TSS is not found at the other end of genes, in the TES.

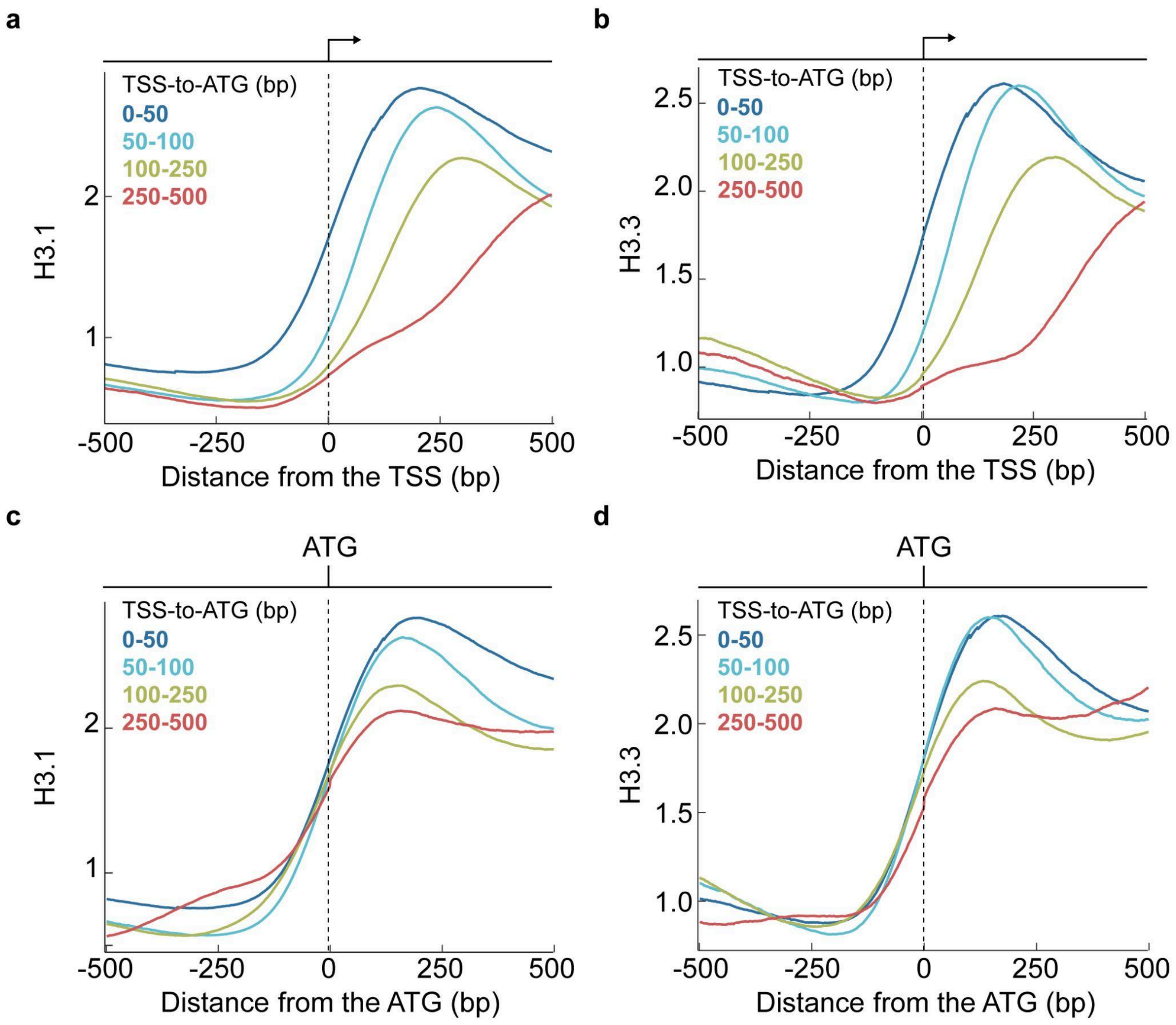

Supplementary Figure 3: TSS-to-ATG distance influences histone H3 enrichment near the TSS

Average enrichments of histones H3.1 **(a, c)** and H3.3 **(b, d)** near the TSS **(a-b)** or ATG **(c-d)** of genes with varying TSS-to-ATG distances. Processed data for enrichment of the two histones along the genome were retrieved from the plant chromatin state database (PCSD)[4], and data from replicates were averaged [5,6].

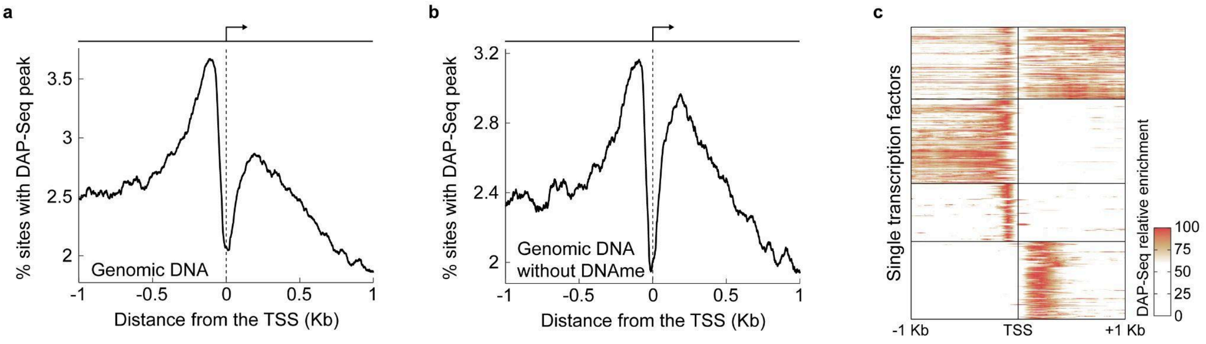

Supplementary Figure 4: TFs bind bimodally both up- and downstream of the TSS (DAP-Seq)

(a-b) Fraction of sites with a DAP-Seq peak center for TFs, as in Fig. 1c, separately plotted for TFs binding purified genomic DNA **(a)** or to non-methylated genomic DNA **(b)**; data smoothed using a 100 bp rolling window. TFs binding downstream to the TSS were more sensitive to DNA methylation. **(c)** DAP-Seq peak enrichment, as in Fig. 1d, for each TF separately, maximum signal for each TF was scaled to 100, enrichment patterns were clustered using k-means (k = 4).

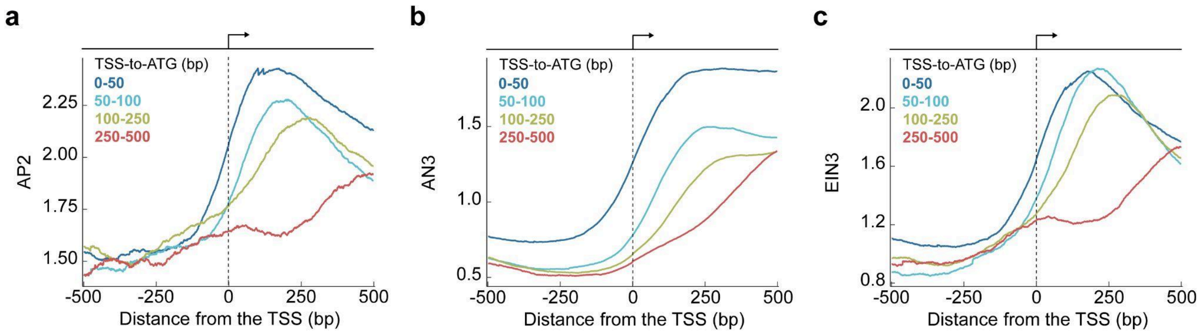

Supplementary Figure 5: In vivo evidence of TFs binding downstream of the TSS

Average enrichment of **(a)** the DNA-binding protein APETALA 2 (AP2), **(b)** the transcriptional coactivator ANGUSTIFOLIA3 (AN3), and **(c)** the DNA-binding protein ETHYLENE INSENSITIVE3 (EIN3) near the TSS of genes with varying TSS-to-ATG distances. Data were retrieved from PCSD, averaged for replicates if available, and plotted as in Supplementary Figs. 3a,b [4,7–9].

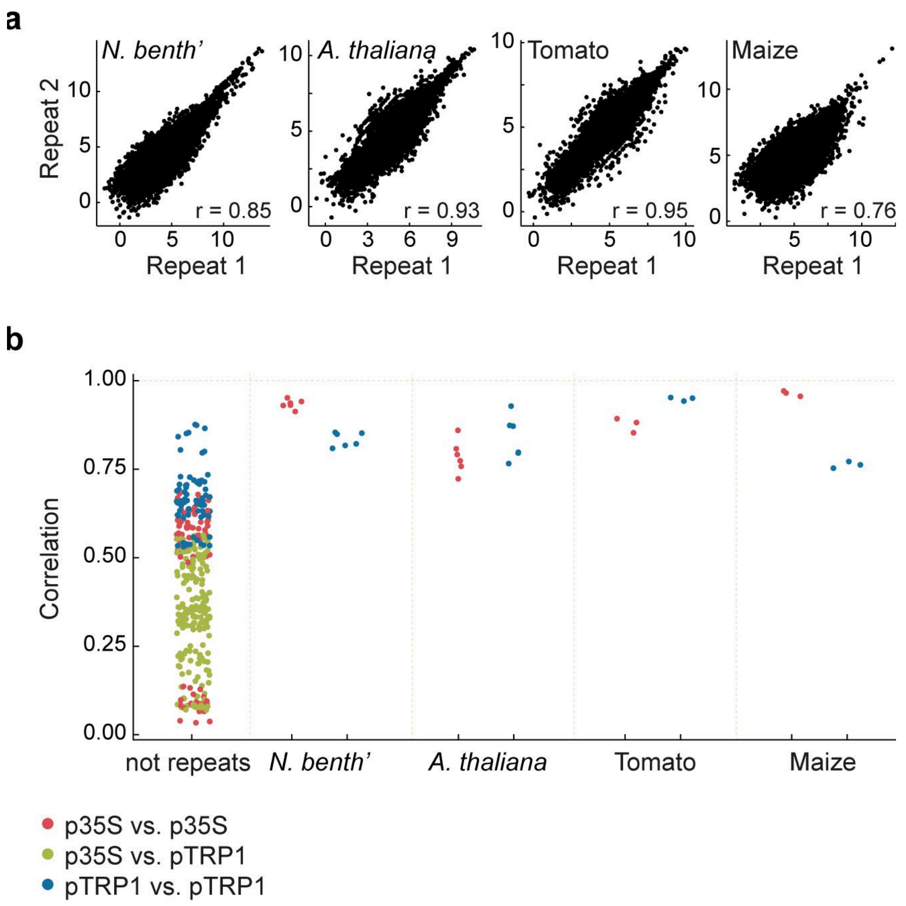

Supplementary Figure 6: Reproducibility of MPRA across four flowering plants

MPRA experiments were conducted in four (*A. thaliana* and *N. benthamiana*) or three (tomato and maize) replicates, with both p35S- and pTRP1-based libraries. Pearson's correlation coefficients were calculated for comparison of all 24,000 constructs for all possible pairs within the 28 experiments. **(a)** Selected scatter plots are presented for pairs of pTRP1-based replicates for each of the four species, with the respective Pearson's correlation coefficients indicated (*r*). **(b)** The full array of Pearson's correlation coefficients for replicates plotted separately for each species, and compared to correlation values between pairs of non-replicates.

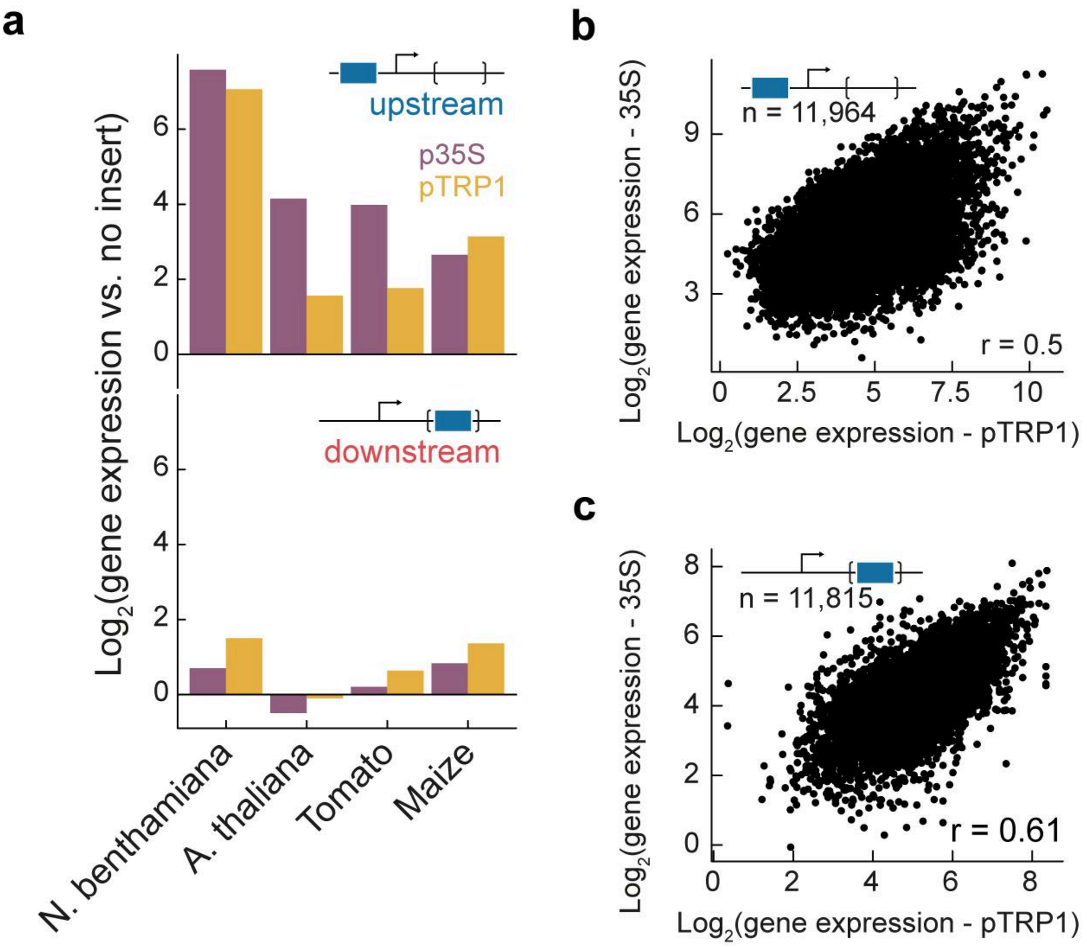

Supplementary Figure 7: Comparison of MPRA results using two core promoters

(a) Shown is the gene expression induced by 35S enhancer fragments vs. constructs lacking insertions in four species within the MPRA framework. Shown is the comparison of gene expression between the two core promoters used in the MPRA: 35S (purple) and the TRP1 gene promoter (orange). The effects are shown for insertion upstream of the TSS (top panel) and downstream of it within an intron (bottom panel). **(b-c)** Shown is the correlation in gene expression across all constructs, across the 12,000 inserted fragments, when comparing the use of the 35S core promoter vs. the TRP1 promoter within the Arabidopsis MPRA. The correlation is shown for insertions upstream **(b)** and downstream of the TSS within an intron **(c)**. Pearson's correlation (*r*) and number of constructs compared (*n*) are indicated in **b** and **c**.

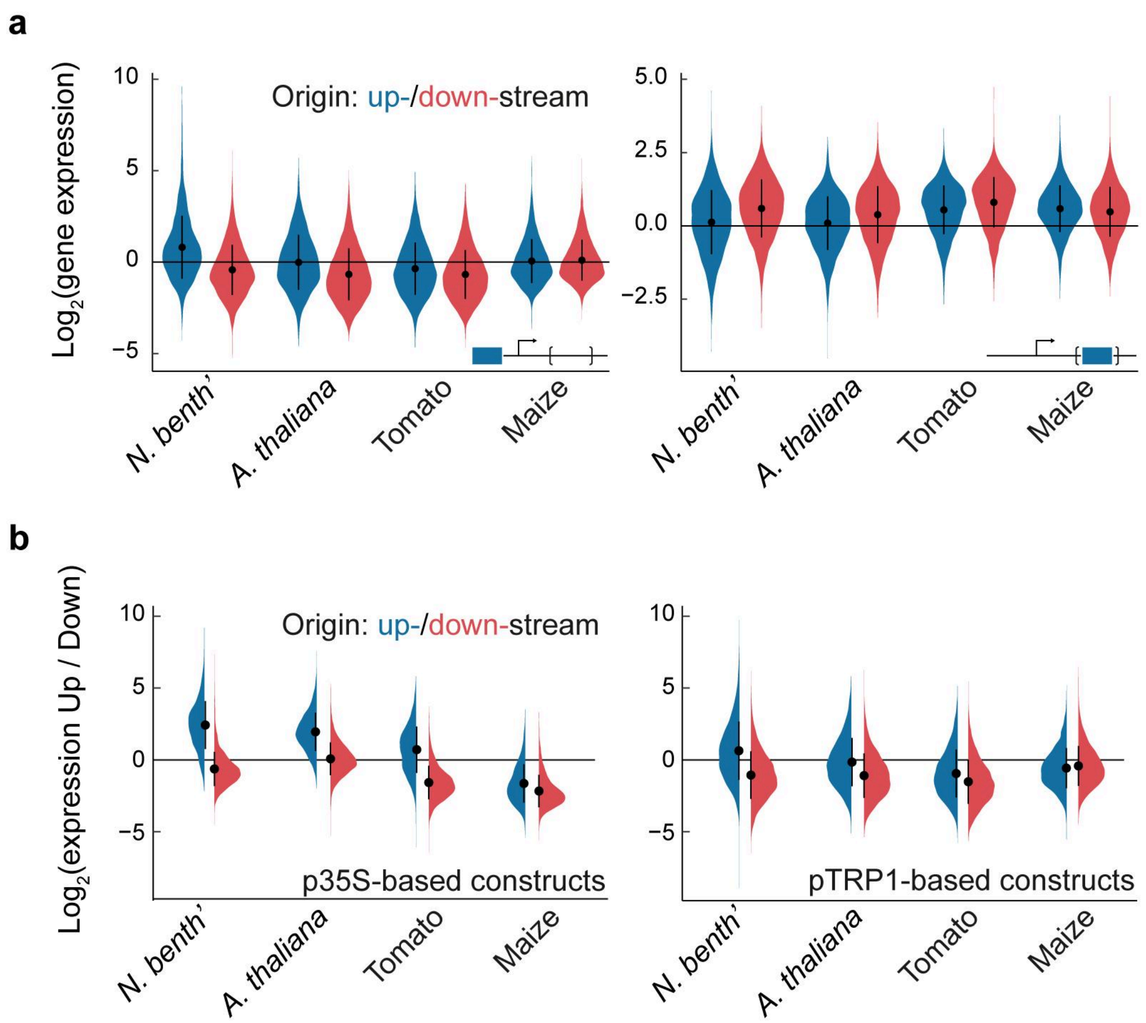

Supplementary Figure 8: Comparative enhancement by upstream vs. downstream originating sequences

(a) Same as Fig. 2f for pTRP1-based constructs. Expression from upstream- (blue, 3415 fragments) and downstream-derived (red, 7843 fragments) fragments is compared against constructs lacking an insertion, with sequences situated upstream (left) or downstream (right). **(b)** Relative expression from identical enhancers due to enhancer position (upstream vs. downstream) separated by fragment genomic origin: upstream (blue, 3,966 or 3,415 fragments for 35s- and pTRP1-based respectively) and downstream (red, 7,928 or 7,843 fragments for 35s- and pTRP1-based respectively), in four different species, for both p35S-based (left) and pTRP1-based (right) libraries. In both A and B, error bars represent the mean and ± standard deviation.

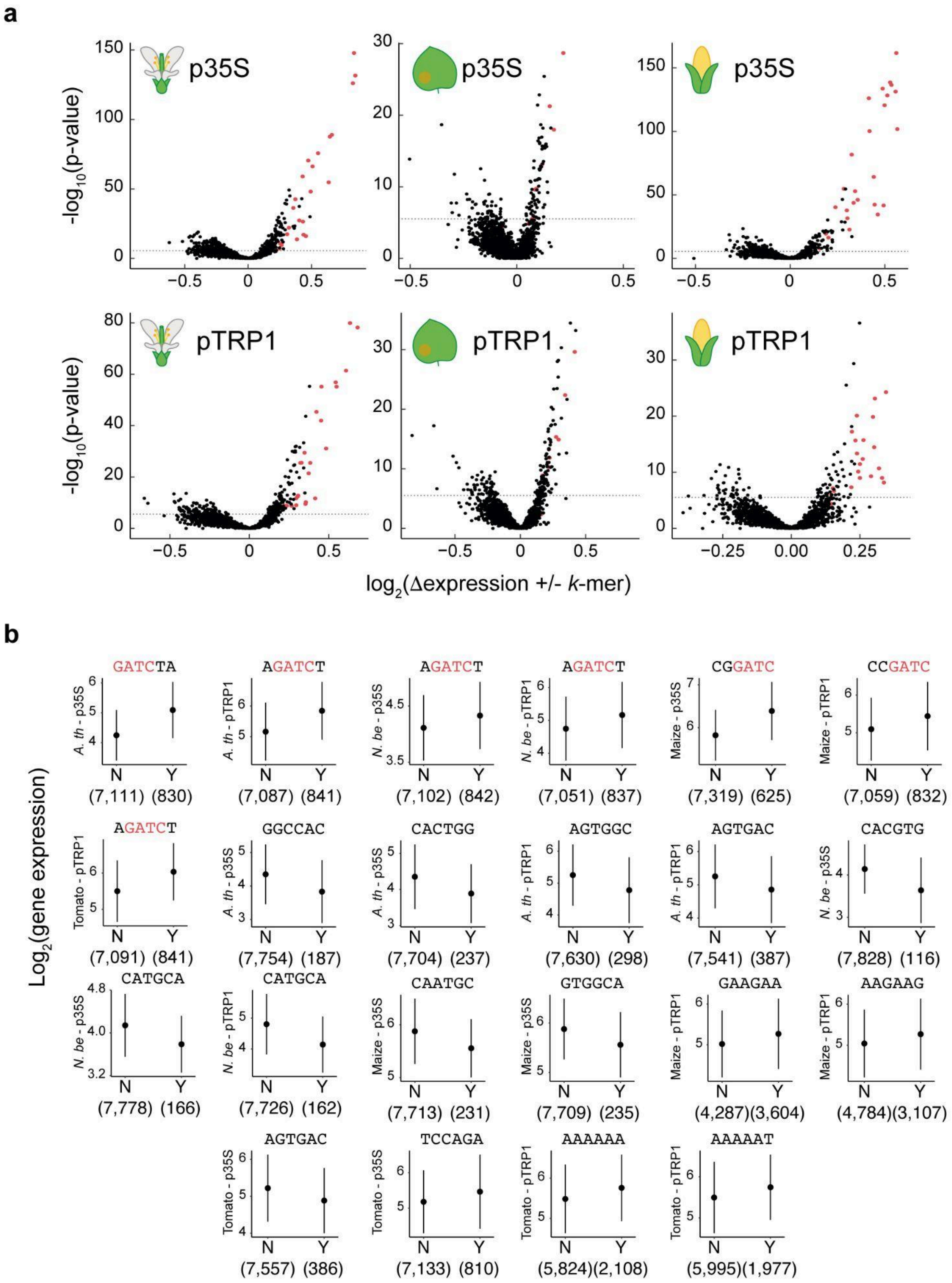
a
p35S
pTRP1
-log10(p-value)
log2(Δexpression +/- k-mer)
b
Log2(gene expression)
GATCTA
AGATCT
CGGATC
CCGATC
GGCCAC
CACTGG
AGTGGC
AGTGAC
CACGTG
CATGCA
CAATGC
GTGGCA
GAAGAA
AAGAAG
TCCAGA
AAAAAA
AAAAAT
A. th - p35S
A. th - pTRP1
N. be - p35S
N. be - pTRP1
Maize - p35S
Maize - pTRP1
Tomato - p35S
Tomato - pTRP1
N Y
(7,111) (830)
(7,087) (841)
(7,102) (842)
(7,051) (837)
(7,319) (625)
(7,059) (832)
(7,091) (841)
(7,754) (187)
(7,704) (237)
(7,630) (298)
(7,541) (387)
(7,828) (116)
(7,778) (166)
(7,726) (162)
(7,713) (231)
(7,709) (235)
(4,287)(3,604)
(4,784)(3,107)
(7,557) (386)
(7,133) (810)
(5,824)(2,108)
(5,995)(1,977)

Supplementary Figure 9: Identifying 6-mers linked to downstream MPRA expression

(a) Score of 6-mers based on their influence on gene expression in the TSS downstream-MPRA, considering only sequences derived from downstream of the TSS in the Arabidopsis genome, as shown in Fig. 3a. For each of the 2,080 unique 6-mers, including reverse complements, downstream-derived sequences were divided into those containing or lacking the 6-mer. A $-\log_{10}$(p-value) from a two-sided Mann-Whitney U test comparing these two groups is plotted on the y-axis, versus the difference in average $\log_2$ expression between the groups containing and lacking the 6-mer on the x-axis. Bonferroni multiple testing 5% threshold (depicted by a horizontal dashed line) is defined as $-\log_{10}$(0.05 / # tests), where the number of tests includes all eight experimental setups. Points in red depict 6-mers containing the sequence GATC. The top row shows p35S-based backbones and the bottom row pTRP1-based ones. Host species from left to right are: *A. thaliana*, *N. benthamiana*, and maize, as indicated by icons. **(b)** Distributions of expression values for downstream-derived fragments with (Y) or without (N) the indicated 6-mer in downstream MPRA, with the number of fragments indicated below the x-axis. Effects of 22 6-mers for specific combinations of backbone x species are shown, similar to Fig. 3b. All 6-mers presented have a p-value smaller than 10^{-12}. The GATC-containing 6-mer with the strongest effect, and the top two GATC-lacking 6-mers for each of the eight backbone-species combinations are displayed, comprising a set of 24 6-mers, which includes the 2 6-mers presented in Fig. 3b. Error bars represent the mean ± standard deviation. Species and backbone are indicated on the y-axis.

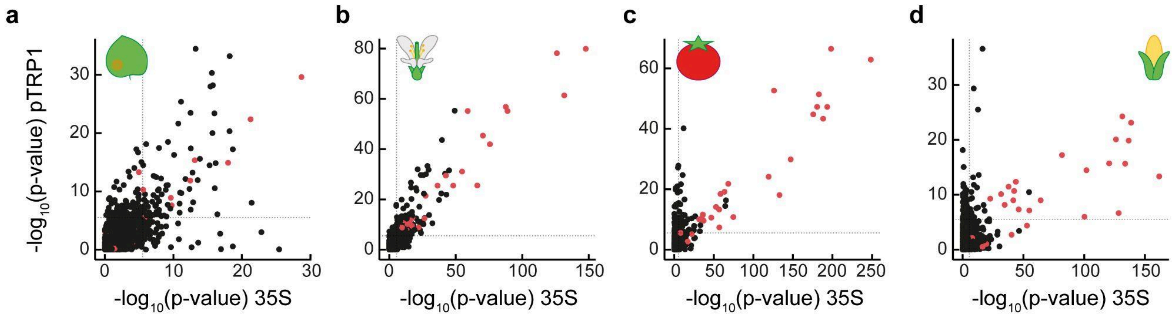

Supplementary Figure 10: Comparison of 6-mers linked to downstream MPRA expression between p35S- and pTRP1-based libraries

Comparison of (-log10) p-values from a two-sided Mann-Whitney U test for 6-mer associations with gene expression, as in Supplementary Fig. 9a, between the p35S- and pTRP1-based libraries. The p-values are shown for MPRA done in the different species: *N. benthamiana* **(a)**, Arabidopsis **(b)**, tomato **(c)**, and maize **(d)**. A dashed line, as defined in Supplementary Fig. 9a, represents the Bonferroni-corrected 5% significance threshold. Points highlighted in red represent 6-mers containing the sequence GATC.

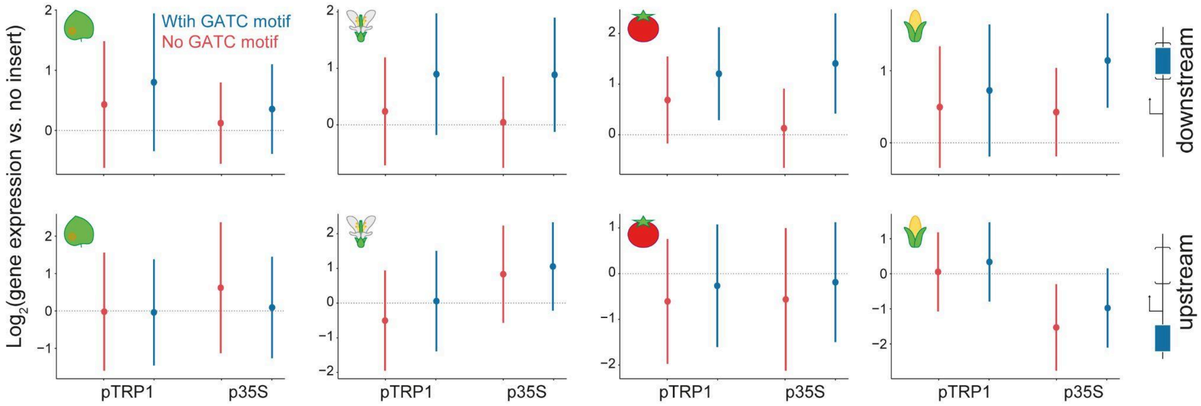

Supplementary Figure 11: Comparison of GATC motif association with gene expression in the upstream and downstream MPRA

Relative activity of all fragments when inserted downstream (upper row) or upstream (bottom row), as a function of the presence of YVGATCBR consensus motifs in the tested fragments. Group sizes: 10,214-10,675 (No GATC motif, red) and 1,281-1,303 (With GATC motif, blue). Backbone and species are indicated. Error bars represent mean ± 1 standard deviation.

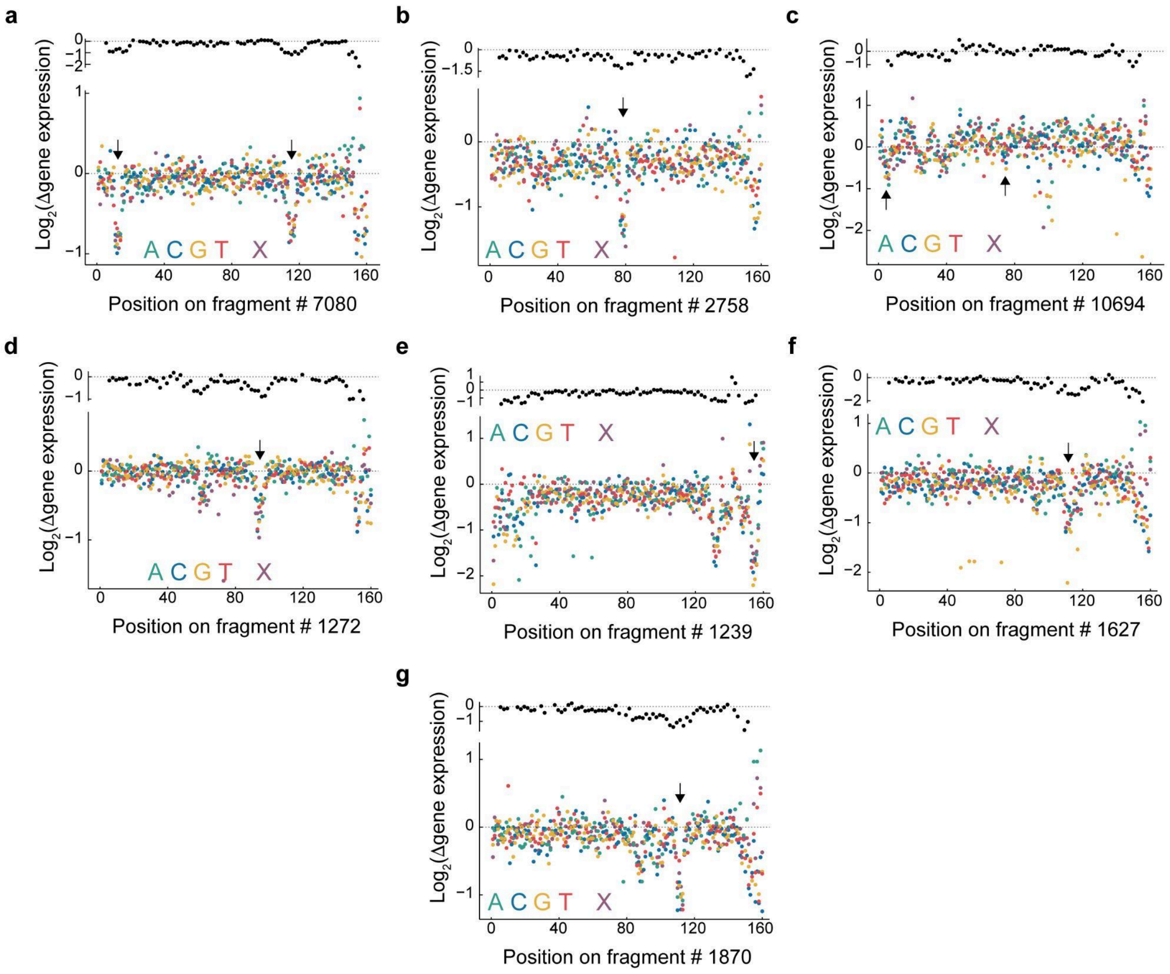

Supplementary Figure 12: Examples of deep mutational scan of fragments in MPRA in pTRP1-based libraries

Deep mutational scans of fragments as indicated in the x-axis using pTRP1-based libraries. All examples presented as in Fig. 4b.

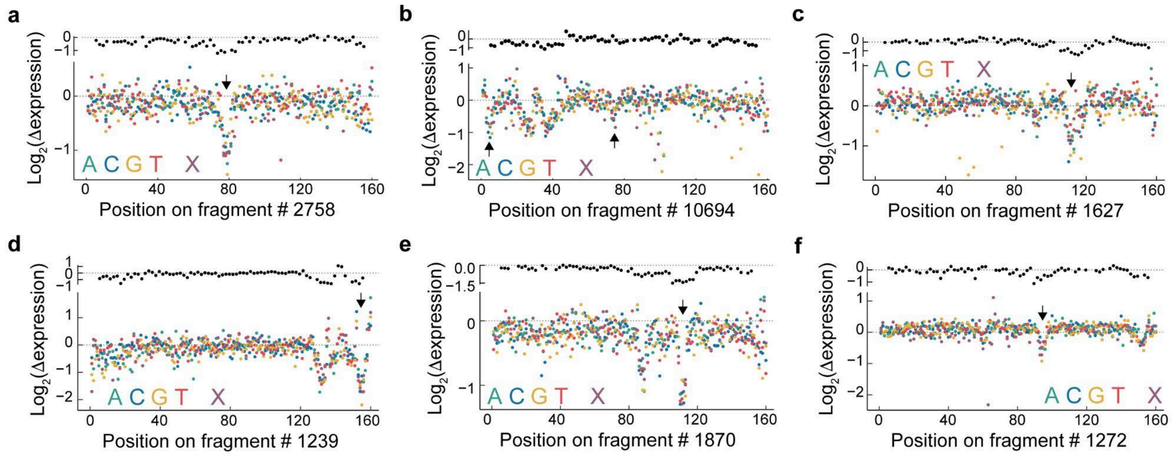

Supplementary Figure 13: Examples of deep mutational scan of fragments in MPRA in p35S-based libraries

Deep mutational scan of fragments as indicated in the x-axis using p35S-based libraries. All examples presented as in Fig. 4b for six additional fragments.

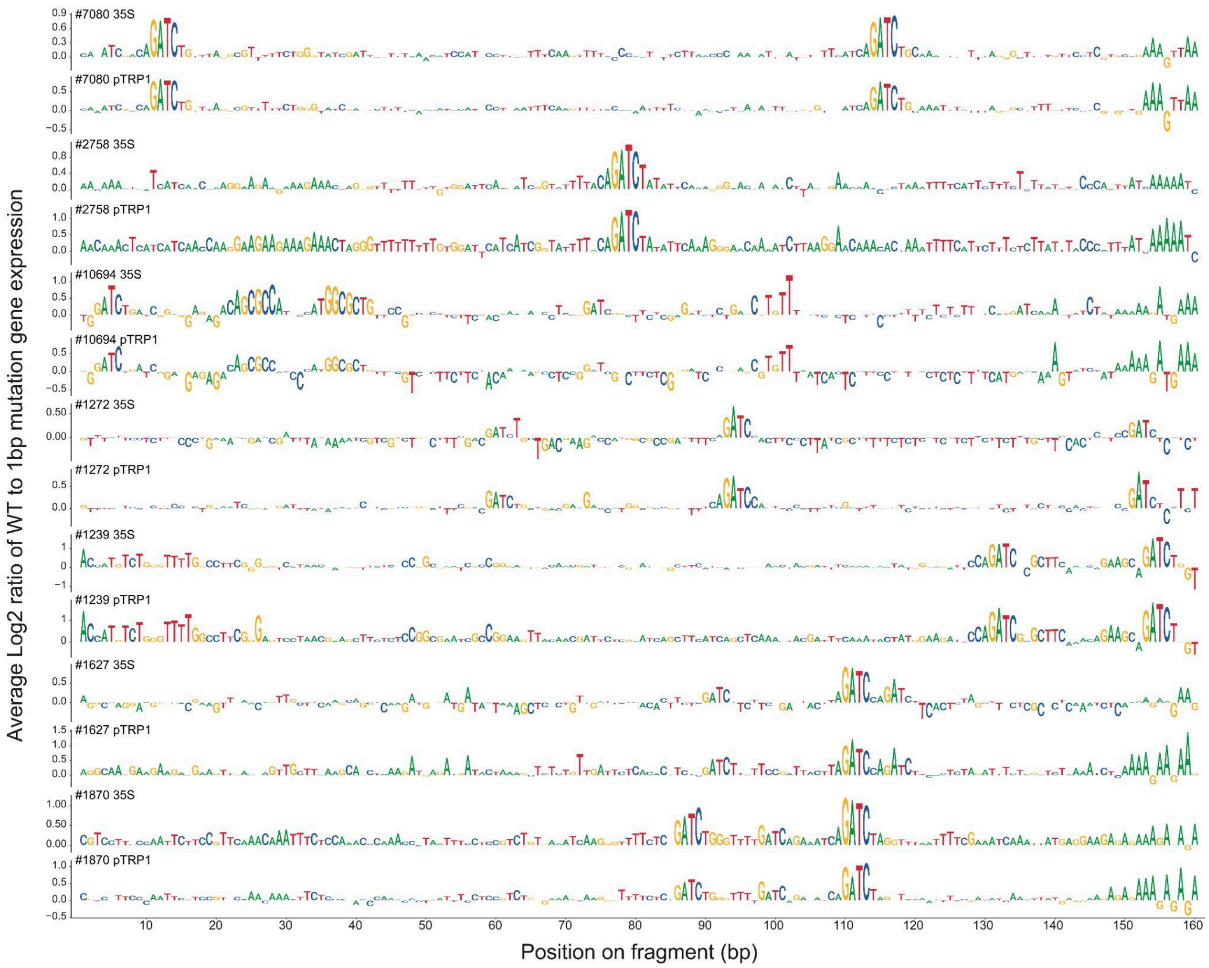

Supplementary Figure 14: The per-nucleotide contribution to transcriptional regulation calculated from deep mutation scanning

The impact of mutating each position within the fragments is shown by plotting the average log2 difference in gene expression between variants with the WT nucleotide and all four of their mutated counterparts. For each 1-bp mutation analyzed, we calculate this difference and plot the average change in expression as the height of the corresponding nucleotide in the original fragment. The plots include the fragments shown in Figs. 4b and Supplementary Figs. 12-13. Each plot is labeled with the fragment index and the library core promoter used.

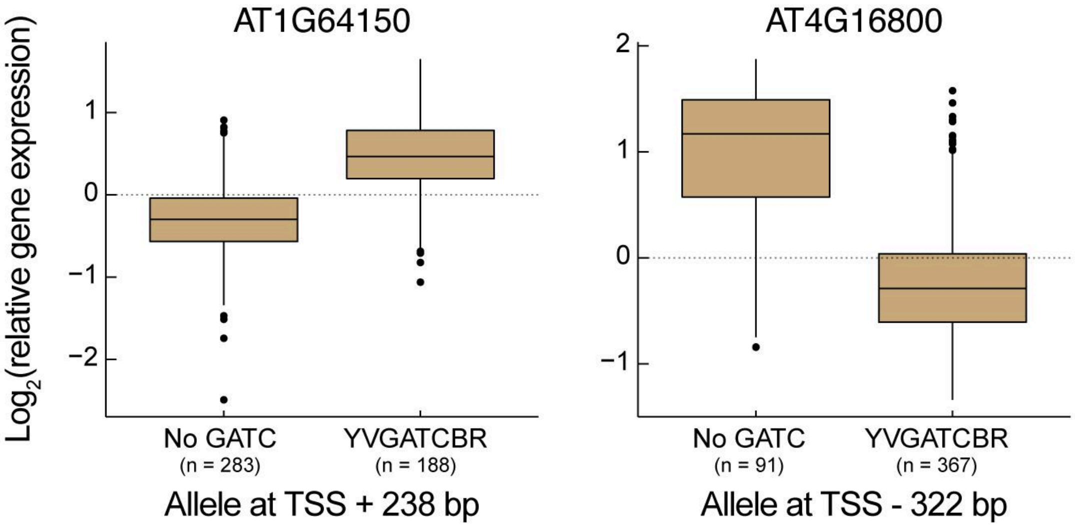

Supplementary Figure 15: Examples of differential gene expression associations with alleles having or lacking a GATC motif in *A. thaliana* natural accessions

Two representative examples from the summary statistics in Fig. 4d, illustrating the association between gene expression and the presence of a GATC motif [1,10]. On the left, accessions with different alleles of AT1G64150 are grouped based on the presence of the GATC motif 238 bp downstream of the TSS or the complete absence of the 4 bp GATC sequence. Intermediate cases are omitted. Expression values are shown relative to the mean in the population. On the right, a similar graph is depicted for gene AT4G16800, with the motif 322 bp upstream of the TSS. Boxplots display the median (center line), IQR (box bounds), whiskers (min and max within 1.5 IQR), and outliers (points beyond whiskers). The number of accessions (n) in each box is shown below the variant type.

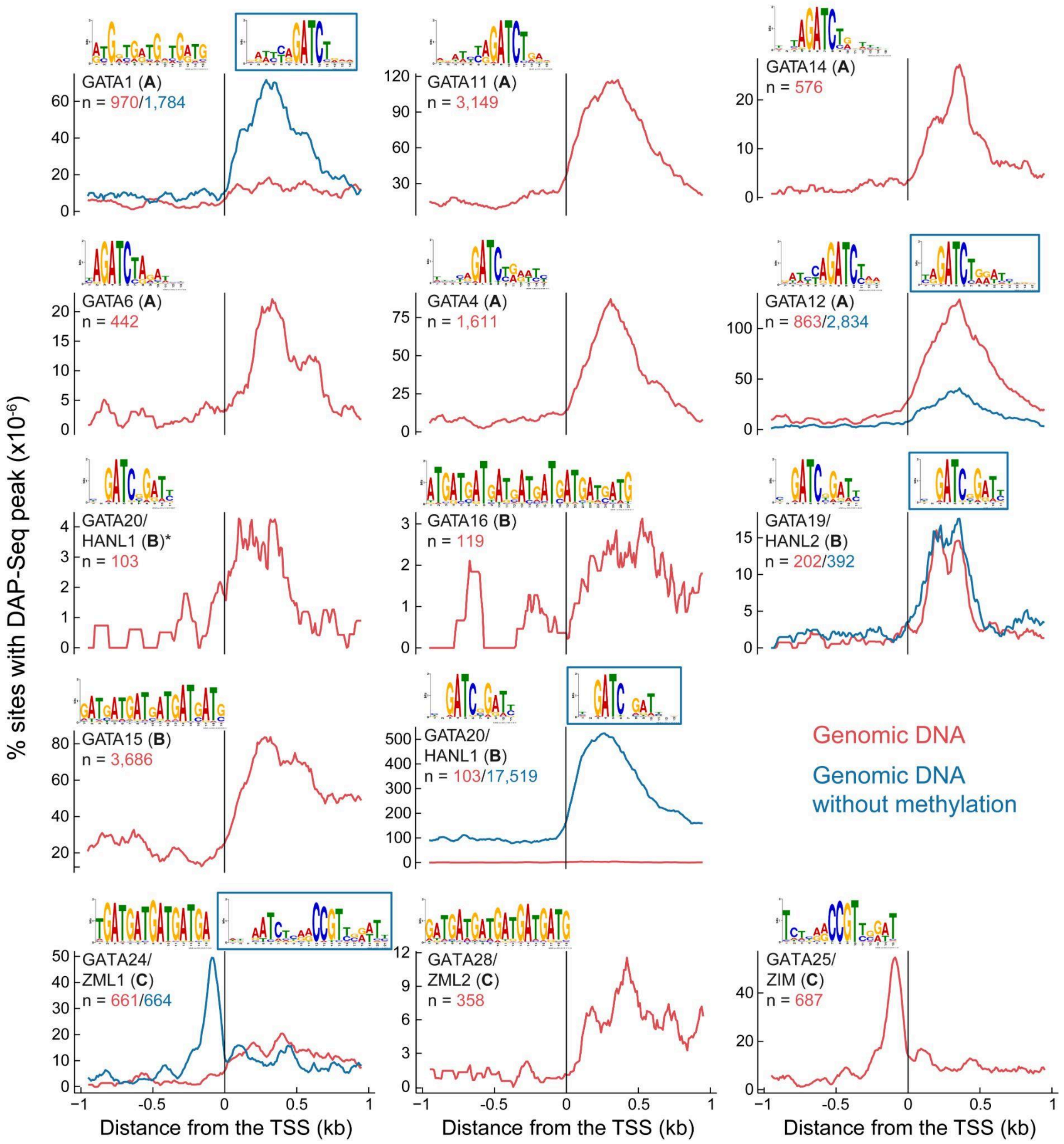

Supplementary Figure 16: DAP-Seq data of GATA TFs from O'Malley et al.

Enrichment relative to the TSS of the binding peaks of each of the 13 GATA TFs examined in ref' [11]. Each GATA TF is plotted in a different subplot as indicated, the subfamily of the GATA is indicated in brackets [12,13]. The number of identified peaks (*n*) is given below the TF name, if the TF was also assayed with non-methylated DNA (blue line), the number of binding peaks for this is also given in blue. Finally, the DNA motif identified in [11] is plotted above the graph, a blue frame is added if it was derived from the assay with non-methylated DNA. GATA20 is plotted twice, once with only the methylated DNA, due to the large difference in dynamic range between them. Note that the DNA motif associated with all of subfamily A is similar to the GATC motif identified in this work, while a few TFs in subfamily B also bind a motif with a GATC sequence, it also has a different affinity for “GAT” not inferred in the MPRA results of this work.

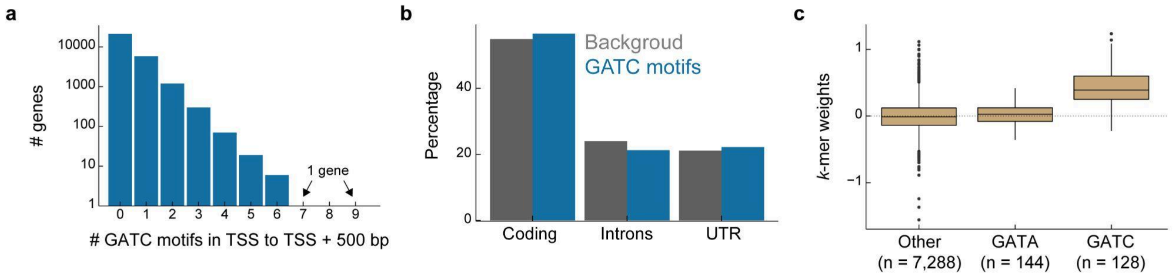

Supplementary Figure 17: Genomic distribution of the GATC motif and its association with GATA factor binding

(a) Distribution of GATC motifs in the 500 bp region downstream of the TSS in Arabidopsis genes. The graph depicts the number of genes with different counts of GATC motifs within this window. **(b)** Distribution of GATC motifs in different genomic contexts within the 500 bp region downstream of the TSS, against the genomic background. **(c)** *k*-mer weights derived from an analysis of GATA12 ChIP-Seq in maize [14]. A higher weight indicates stronger binding propensity of the transcription factor GATA12 to sequences containing that specific *k*-mer. The weights of *k*-mers containing either GATC or GATA are plotted along weights of all other *k*-mers. The weights serve as a measure of sequence preferences when modeling GATA12 TF binding based on the machine-learning model applied by the authors. The comparison to GATA-containing *k*-mers is highlighted due to the propensity of GATA factors to bind GATA sequences in other species (Supplementary Note 1). Boxplots display the median (center line), IQR (box bounds), whiskers (min and max within 1.5 IQR), and outliers (points beyond whiskers), numbers of *k*-mers (*n*) are indicated.

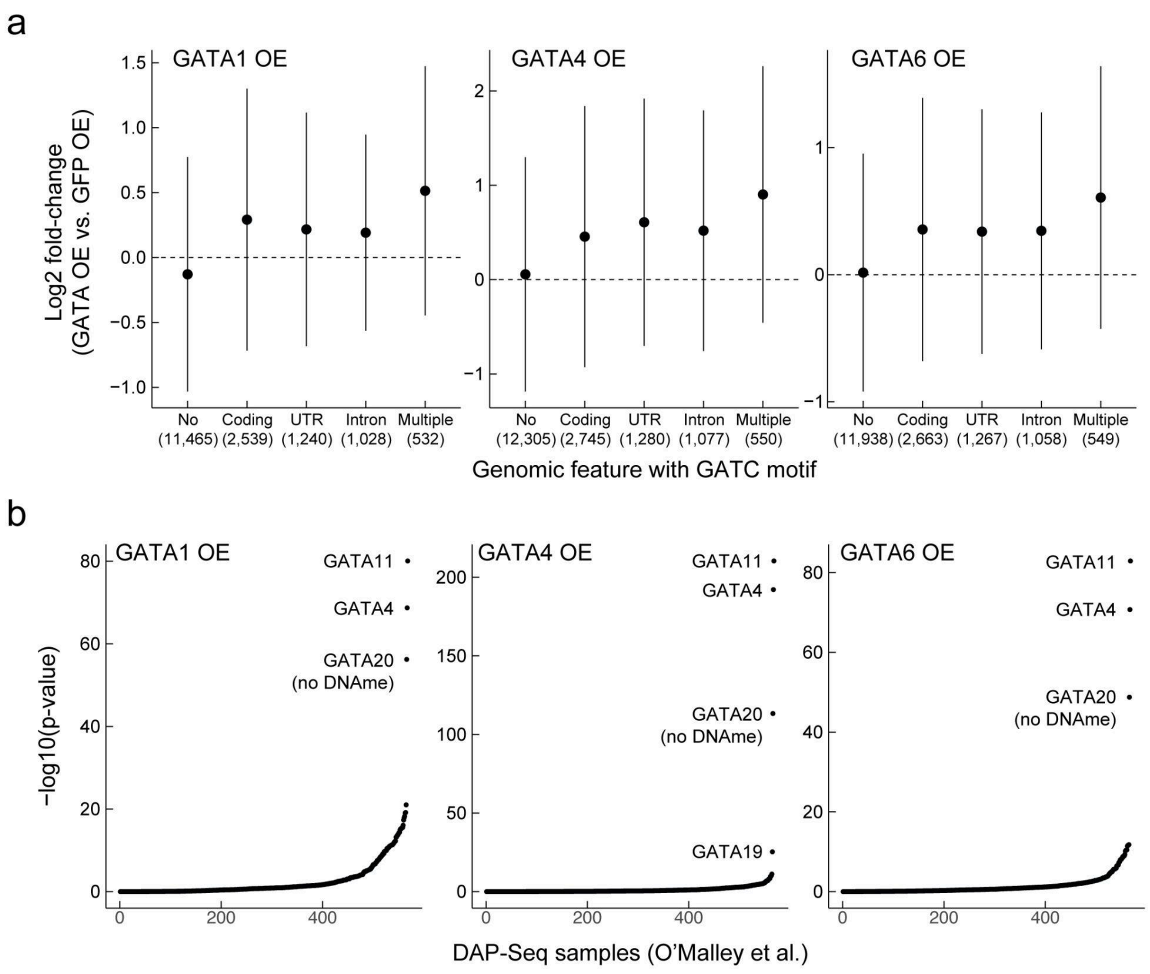

Supplementary Figure 18: Transient overexpression of GATA TFs affects genes identified as GATA targets by DAP-Seq

(a) Shown is the log2-fold change in gene expression in response to GATA TFs OE (as indicated) relative to GFP OE. Data are plotted for genes without GATC motifs in the 500 bp downstream of the TSS, genes with GATC motifs only in coding regions, UTRs, introns, or those with motifs in more than one genomic feature. Error bars represent the mean ± 1 standard deviation; numbers of genes per category are indicated. **(b)** Enrichment analysis was performed to compare gene targets identified by DAP-Seq for all TFs assayed in the [11] study with genes upregulated (as defined in Extended Data Fig. 7c) upon overexpression of the GATA TFs. Enrichment p-values were calculated using an upper-tail hypergeometric test and are shown for each of the TFs samples previously assayed by DAP-Seq [11]. For the three GATA overexpression experiments, the genes with increased expression are most enriched with the targets of GATA TFs in the DAP-Seq data.

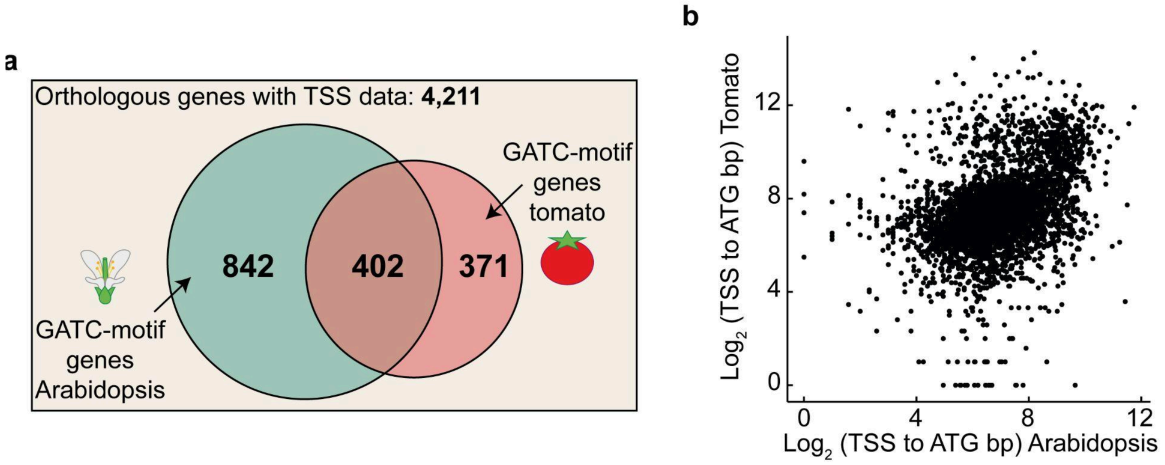

Supplementary Figure 19: Conservation of GATC-motif genes between Arabidopsis and tomato

One-to-one orthologous genes between Arabidopsis and tomato were identified using OrthoFinder [15]. Out of 6,883 one-to-one pairs, 4,211 possessed a TSS upstream of the ATG in both species, forming the basis for comparison. **(a)** The conservation of genes containing a GATC motif within the 500 bp region downstream of the TSS is depicted. The Venn diagram illustrates orthologous genes and those harboring the GATC motif in one or both species. The intersection of genes possessing the motif is statistically significant (p-value < 10^{-49}) according to an upper-tail hypergeometric enrichment test. **(b)** Analysis of the distance between the TSS and the ATG demonstrates conservation between Arabidopsis and tomato, with a Spearman correlation of 0.42 and a Pearson correlation of 0.26. Notably, this level of correlation, though slightly stronger here, parallels findings from an earlier study comparing the 5' UTR length between *Candida albicans* and *Saccharomyces cerevisiae* [16].

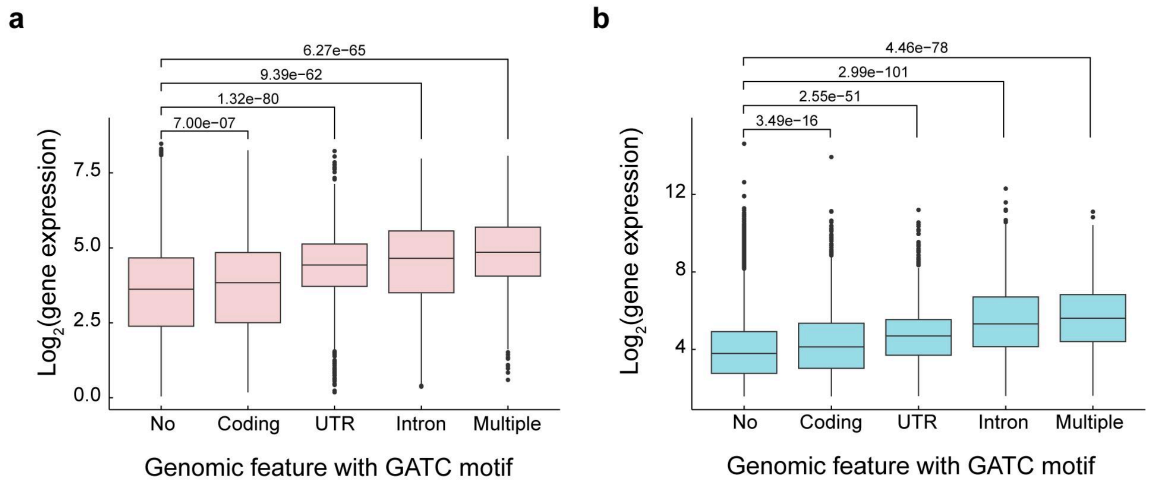

Supplementary Figure 20: Association between GATC-motifs presence and gene expression across different genomic contexts

Gene expression levels are displayed for the aerial parts of Arabidopsis seedlings [17], as depicted in Fig. 5a **(a)**, and in seedling roots [18], corresponding to Fig. 5f **(b)**. Genes are grouped according to the GATC motifs located within the 500 bp region downstream of the TSS, categorized by specific genomic features: no GATC motifs in this region (21,227 genes), motifs exclusively in the coding region (4,003 genes), in UTR regions (1,395 genes), in introns (1,271 genes), and genes with GATC motifs spanning more than one feature type, such as one motif in the coding region and another in a UTR (600 genes). p-values are shown for comparison of gene expression levels for genes with GATC motifs in any of the feature categories versus those without any GATC motif within the 500 bp region downstream of the TSS. Boxplots display the median (center line), IQR (box bounds), whiskers (min and max within 1.5 IQR), and outliers (points beyond whiskers). Statistical significance was assessed using the Welch two-sample t-test.

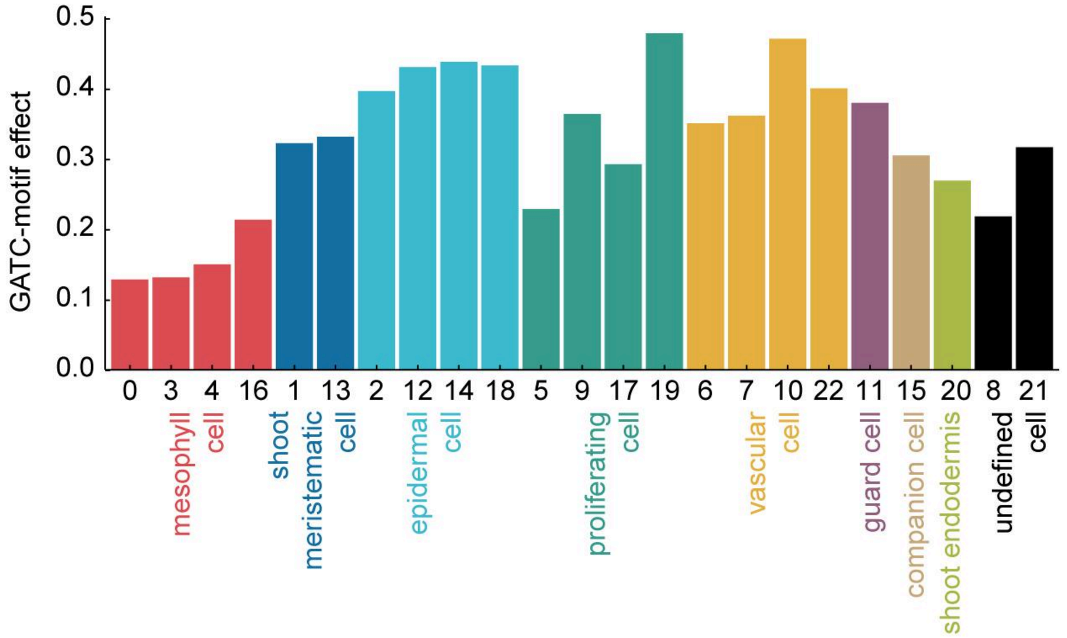

Supplementary Figure 21: Effect of GATC motif on expression throughout the Arabidopsis vegetative shoot

GATC-motif effect sizes across different cell types within the Arabidopsis vegetative shoot, as quantified in Fig. 5g. Gene expression was determined by averaging from scRNA-seq data, categorized based on the 23 clusters defined in ref. [19]. Clusters are annotated as presented in the original study.

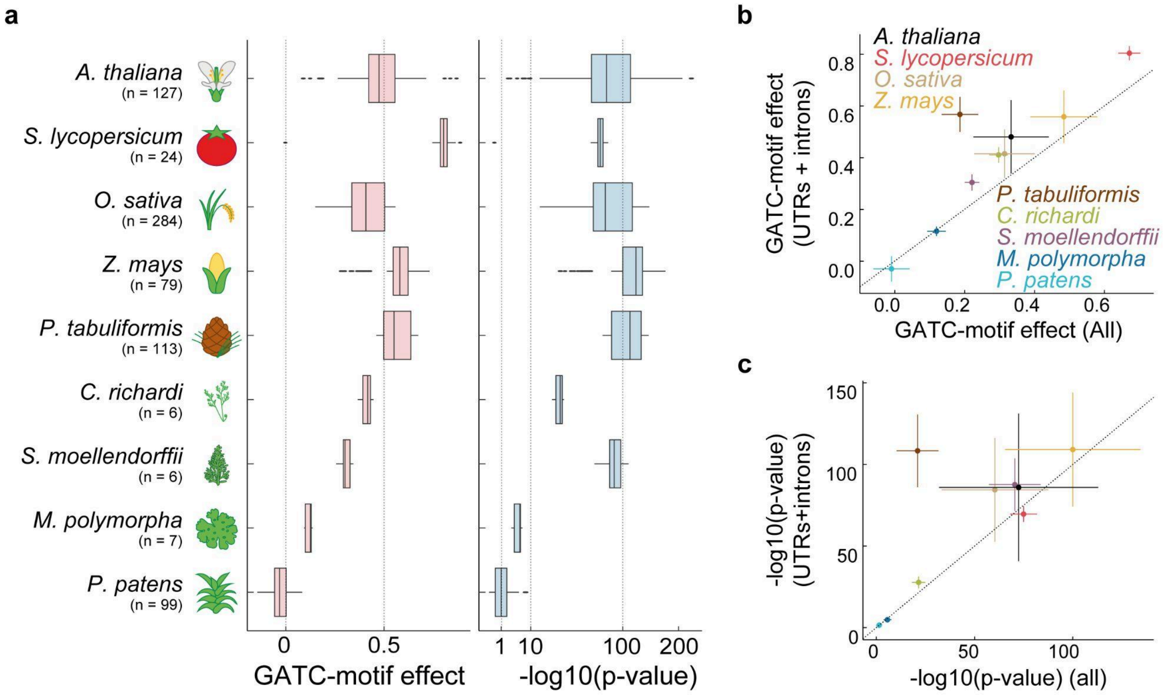

Supplementary Figure 22: Downstream GATC motifs are associated with higher gene expression when located in introns or UTRs across species

(a) Association between GATC-motif downstream of the TSS and gene expression across land plant species is shown as in Fig. 6, counting only motifs found either in introns or UTRs in the 500 bp downstream of the TSS. The effect of the GATC motif on expression (slope, left panel) and the significance of the association (two-sided t-statistic p-value, right panel) are shown. Boxplots display the median (center line), IQR (box bounds), whiskers (min and max within 1.5 IQR), and outliers (points beyond whiskers). The right panel x-axis is square-root scaled. **(b-c)** Comparison of GATC motif effect size **(b)** and significance of associations **(c)** considering GATC motifs found in all genomic contexts (x-axis) vs. only ones in introns or UTRs (y-axis). Error bars represent one standard deviation around the mean. Points are coloured according to their species, as described in the legend of **b**. Dashed lines in **b** and **c** mark y=x. The number of transcriptomic datasets used for each species is indicated in **a** (below the species name).

Supplementary Note 1 - Indications of plant GATA factors binding to the GATC Sequence

GATA transcription factors were first studied in erythrocytes [20,21]. Despite their name, derived from their ability to regulate transcription via G-A-T-A DNA sequences, some of these factors often bind non-GATA sequences. Sometimes with even higher affinity than their namesake sequences. Notably, a subset of GATA transcription factors have repeatedly been found to bind the G-A-T-C sequences.

Evidence of this can be seen in various species. Vertebrate GATA-1, GATA-2, and GATA-3 have demonstrated binding to GATC in addition to GATA sequences [22–24]. In C. elegans, the ELT-1 GATA transcription factor showed a significantly stronger transcriptional activation effect on GATC sequences compared to GATA sequences [25]. Similarly, in the mushroom *Coprinopsis cinerea*, the CcNsdD2 GATA TF showed a preference for the GATC sequence [26]. Also in the well studied budding yeast (*S. cerevisiae*) four out of nine GATA TFs are associated with a GATC motif and not a GATA one [27].

In 2000, a study by Lowry & Atchley analyzed GATA protein sequences across multiple species [28]. They found that GATA TFs from *A. thaliana* grouped separately from vertebrate GATAs. Subsequent large-scale studies investigating TF-binding motifs, both cross-kingdom and Arabidopsis-specific, profiled 19 GATA TFs from Arabidopsis [11,29,30]. Interestingly, in these studies, none of the Arabidopsis GATA TFs showed binding to a GATA motif, but in most cases rather demonstrated a preference for the GATC sequence (Supplementary Fig. 16).

Further support for the preference for the GATC sequence emerges from more focused studies in plants. ChIP-Seq analysis of the GNC and CGA1 GATA TFs in Arabidopsis, for instance, showed binding to the GATC motif [31]. Similarly, ChIP-Seq in maize identified a preference for GATC, rather than GATA sequences, for the GATA12 TF [14] (Supplementary Fig. 17c).

Evidence from other plant species also reflects this trend. In tobacco, the AGP1 GATA TF was found to induce expression by binding a GATC-containing motif [32]. In *Catharanthus roseus*, the CrGATA1 GATA TF activated five light-responsive vindoline pathway genes via the GATC motif. Intriguingly, removing the GATA sequence did not affect this induced expression [33].

Taken together, these findings from both large-scale and gene-specific studies suggest that, in a majority of instances, GATA TFs in plants primarily interact with GATC motifs.

Supplementary Methods:

Processing gene expression data from the *A. thaliana* accessions

Raw RNA-Seq data from ref. [1] were downloaded from NCBI's Sequence Read Archive (SRA) database, accession SRP074107. The data were separated into two main batches, done more than a year apart, as communicated by the authors of the original study. To verify accession identity, SNPmatch (v5.0.1) was used[34]: briefly, single nucleotide polymorphisms (SNPs) were called for each RNA-Seq sample against the reference genome (TAIR10), and compared to the SNPs of the 1,001 Genomes Project[10]. In a few cases a mismatch between the indicated accession and the identified accession was found. Most of these cases intersected with known mix-ups in the 1001 Genomes Project[10,34]. These mix-ups were corrected, or in ambiguous cases, data were not used. Reads were trimmed using Trim Galore with default parameters[35]. Next, gene expression was quantified for all accessions which are part of the 1,001 Genomes collection, leaving out 44 accessions unique only to the gene-expression dataset. To quantify gene expression, a pseudo-genome was created for each accession by incorporating SNPs from the 1,001 Genomes vcf file, using bcftools (v1.16) consensus option on the reference genome (TAIR10)[36]. Gene expression per sample was quantified using STAR (v2.7.9) against the accession's pseudo-genome using the Araport11 annotations[37,38]. 72 samples with fewer than $4*10^6$ sequencing reads were omitted from further analysis. After discarding the chloroplast and mitochondria genes, gene expression was calculated by dividing the read count by gene length and normalizing to a total signal of 10^6. Then, genes with signal less than 2 were discarded, and gene expression was log_2 transformed.

In a subset of the RNA-Seq libraries, a gene length bias was identified, where coverage was reduced at the 5' end of the genes. To address potential biases associated with gene length, a normalization strategy using a custom R script was implemented. Each batch was processed individually and the difference in gene expression between all possible sample pairs was calculated and correlated to the log_2-transformed lengths of genes. Subsequently, for each sample, the average absolute correlation across all pairs involving that sample was computed. The 10%

of samples with the lowest average correlation were designated as the background group, as they were least affected by the gene length bias. These samples were left unchanged. For each non-background sample, the linear fit for the differences relative to the log_2-transformed gene lengths were determined when compared with each background sample. The average slope from these linear fits served as the correction factor. By subtracting the product of the correction factor and the log_2-transformed gene lengths to the respective samples, the gene length bias was corrected. Lastly, the total gene expression per sample was adjusted to be 10^6 after the correction.

Raw RNA-Seq data from ref. [2] were downloaded from NCBI's SRA, accession SRP036643. Data of 12 samples (6 accessions x 2 conditions) were not part of the SNPs 1,001 Genomes dataset, and were omitted, as well as 46 samples with less than 10^6 reads. Gene expression was quantified as above, first by trimming the sequence reads using Trim Galore and then quantifying using STAR against the pseudo-genomes, calculating TPMs per gene.

eQTLs analysis

The 1,001 Genomes vcf file was filtered for minor allele count >=5 using vcftools (--min-alleles 2 --mac 5) and converted to Plink binary format[39] using plink (v1.9). The Kinship matrix was calculated according to EMMA, using the *k*-mers-GWAS implementation with default parameters[40,41]. For running the eQTL analysis on data from ref. [1], the analysis was conducted on the two batches separately. Only genes with values for at least 200 accessions were used. Gene expression (log_2 transformed, as described above) was normalized by subtracting the average signal of the gene in the batch, averaged over repeats if present, and transforming using a Box-Cox transformation by the MASS R library[42]. Transformed values were used to run Genome-wide associations (GWA) with linear-mixed-models using the kinship matrix by GEMMA (v0.98.5, -lmm 2, -maf 0.05). Only SNPs up to 10 Kb downstream or upstream of the gene were used.

The eQTL analysis for data from ref. [2] was done for the two conditions (10°C and 16°C) separately. For each one, a gene was used if it had values for at least 120 accessions. Normalization of gene expression and following GWA was done as for the dataset from ref. [1]. data. For each of the 4 eQTL analyses done, a threshold for significant SNPs was defined as 0.05 divided by the total number of SNPs used in the analysis, on all genes. These thresholds varied between $1.59 * 10^{-8}$ to $1.16 * 10^{-8}$,

or 7.80 to 7.93 in $-\log_{10}$, between the four analyses. 2760, 4259, 335, and 304 genes had at least one SNP that passed the threshold for batch1, batch2 of the data from ref. [1] and 10°C and 16°C conditions for data from ref. [2], respectively. Analysis presented in the main text (Fig. 1a,b) are from the second batch of ref. [1] as well as eQTL enrichment for different groups of genes (TSS-to-ATG distances or exonic fraction in 500 bp downstream of TSS), results from the other eQTL analysis is presented in the Extended Data Fig. 1 and Supplementary Figs. 1,2.

For plotting the average enrichment of eQTLs relative to the TSS or the transcription end site (TES), each gene with significant associations had the same total contribution to the analysis, regardless of the number of associated SNPs. Two methods to weight the different associated SNPs per gene were used. First, each significant SNP got equal weight. Second, significant associated SNPs were scored according to the posterior inclusion probabilities (PIP), which take into account the linkage disequilibrium (LD) between them. To calculate the PIP, fine map analysis was conducted using the SusieR (v0.12.35) library[43]. For the fine-map analysis a previously calculated imputed matrix of the 1,001 Genomes SNP matrix was used[44]. For each GWA run, imputed genotypic information for the significant SNPs were extracted and PIP were calculated using the phenotype as used for the GWA.

For eQTL analysis of data from ref. [3] the eQTL analysis results from the original study were used. The p-values from the common effect were used with the same threshold used (10^{-7}). Significant SNPs per gene were weighted equally.

Metaplots of genomic profiles around the TSS of genes

To create a plot for genomic data around the TSS of genes, the following strategy was employed to account for overlapping and nearby genes. First, each genomic position was used at most once. For tail-to-tail genes, every upstream position was assigned to the nearest TSS. If a position was both upstream of a gene and within another gene, it was allocated to the gene it was part of, and therefore considered downstream of the TSS. If a position was located inside multiple genes, it was assigned to the TSS that was closest. The relationship between genomic positions and their corresponding TSS was stored and subsequently used to generate an average signal plot surrounding the TSS for a selected group of genes. Genomic annotations were taken from TAIR10. Analysis and plotting of genomic information was done using ‘misha’ and ‘tidyverse’ R packages[45]. Plotting genomic data around the start codon (ATG) of genes was done using the same procedure.

Nucleotide diversity (pi) per position in the genome was calculated using vcftools (v0.1.16) with --site-pi parameter on all the SNPs table of the 1,001 Genomes project[46].

ChIP-Seq data from the plant chromatin state database (PCSD) was downloaded in bigwig format and imported into a misha database in R[4].

ChIP-Seq data for CGA1 was obtained in two independent repeats from the SRA database[31]. Using Bowtie2, these reads were aligned to the TAIR10 reference genome with standard parameters[47]. Subsequent peak calling was performed with MACS2 (v2.2.7.1), employing the 'callpeak' option and parameters set to '-B -q 0.01', allowing for the generation of a genomic profile[48]. For downstream analyses, these genomic profiles were loaded into the misha database.

In the analysis of DNA Affinity Purification and Sequencing (DAP-Seq) data, narrowPeak files were used to identify the genomic positions of TF-binding peaks. These files were from the NCBI's GEO database, accession number GSE60143[11]. Using this data, various genomic tracks were constructed, each capturing the central points of the respective peaks. Distinct tracks were generated for each Transcription Factor (TF), done separately for each type of DNA source - genomic DNA and genomic DNA without DNA methylation. In addition, tracks were created that encompassed all peaks associated with a given TF family. This method was only applied to TF families with a minimum of 10 members. Furthermore, tracks that included all peaks linked to any TF were assembled, separate tracks were prepared for each DNA source, and one for the full dataset combining both DNA sources.

A genomic track for the GATC-motif (YVGATCBR) was constructed within a misha database. In this track, all central positions of the motif were assigned a value of 1, while all other positions were set to 0. This genomic track was used to generate the meta-plot surrounding the TSS and to define genes with a GATC motif within the initial 500 bp downstream of the TSS.

Design of oligonucleotide pools

Every fragment in oligonucleotide pool 1 or 2 (OP1 / OP2) was designed to have a core sequence, not longer than 160 bp surrounded by AGTTCAAACGGTCTCCACTC and AGGACGAGACCAATGTGAAC in the 5' and 3' ends, respectively.

Oligonucleotide pool 1 (OP1) was designed to incorporate fragments near the TSS of specific genes, along with control fragments. The upstream control fragments were

based on the 35S, AB80, and RbcS_E9 enhancers; the fragments were taken from ref. [49]. The downstream control fragment was designed according to the intron of the UBQ10 gene, from 3 base pairs (bp) past the donor site to 3 bp before the acceptor site. Fifty oligonucleotides of 160 bp each were designed to cover each control fragment, with an overlap of 150 bp between every two consecutive oligos (for instance, 1-160, 11-170, 21-180, and so on).

The oligos drawn from near the TSS of genes were designed in the following way: Genes of *Arabidopsis thaliana* were selected according to the TAIR10 annotation[50] under certain criteria: (1) excluding genes on the mitochondria or chloroplast genomes, (2) ensuring the closest upstream gene is at least 50 bp away from the gene's TSS, (3) only considering genes with a single TSS, (4) focusing on genes of at least 500 bp in length, (5) only including protein-coding genes, and (6) selecting highly expressed genes with expression in Col-0 of at least 10 in $\log_2$(TPM) in ref. [1].

Three fragments were obtained from the genome for each of the 7,775 genes that passed this filtering process: 200 bp to 41 bp upstream of the TSS, 41 bp to 200 bp and 201 bp to 360 bp downstream the TSS. If any of these fragments contained a donor or an acceptor splicing site, the sequences surrounding it, 5 bp (-1 to +3) for donor sites and 3 bp (-1 to +1) for acceptor sites, were removed. Any genes with a BsmbI or BsaI recognition site within the three TSS-proximate sequences were then removed, reducing the gene count to 4,884. Finally, 3,991 genes with the highest expression were chosen, and the three fragments around each of these genes' TSS were included in the oligonucleotide pool.

Oligonucleotide pool 2 (OP2) was constructed to contain altered versions of fragments from OP1, which were split into three distinct sets. The goal of Set 1 was to mutate the GATC motifs. 823 fragments originating downstream of the TSS in OP1 were chosen, each with at least one GATC motif, adding up to a total of 917 GATC elements. New fragments were created for each of these fragments by either of the following: (1) removing the GATC motif, resulting in shorter fragments, (2) rearranging / shuffling the 8 bp of the GATC element to ensure disruption of the original GATC sequence, and (3) changing the 6th nucleotide in the element, from "C", to an "A".

If a single fragment contained more than one GATC motif, each of these modifications was applied to every possible subset of elements. For instance, if a fragment had three GATC motifs, it would yield seven subsets (2^3-1), and each of the

three transformations would be applied to each of these, resulting in 21 mutated fragments. In total, 3,216 fragments were created for Set 1.

Set 2 was designed with the objective of introducing GATC motifs into the fragments. For this purpose, 221 fragments which can be detected consistently in our MPRA libraries were arbitrarily selected from OP1, with 75% originating downstream and 25% originating upstream of the TSS. For each chosen fragment, an incremental series of 8 fragments was created, with each successive fragment incorporating an additional CAGATCTG sequence. There was a minimum of 2 bp between two adjacent GATC motifs. As a result, a total of 1,768 fragments (221 * 8) were designed for Set 2.

Set 3 was designed for a deep mutational screening of a small subset of fragments. To this end, 20 fragments from OP1, all originating from downstream of the TSS, were selected. The chosen fragments were among the top 100 enhancing fragments from the six libraries of tomato, *A. thaliana*, and *N. benthamiana*, in each of the two backbones when positioned downstream the TSS. Among these 20 fragments, seven were devoid of any GATC elements, from which two fragments lacked any GATC sequences (only the 4 bp). Out of the 13 fragments that did have a GATC element, two of them had two such elements. In the scope of this paper, only these 13 are analyzed, while all data can be found in the Supplementary Tables.

Two series of modifications were created for each of these fragments. First, every alternating 10 bp sequence, that is positions 1-10, 3-12, 5-14,..., and 151-160, was removed, leading to 76 fragments each of 150 base pairs in length. Second, each single bp in the fragment was substituted with one of the other three potential nucleotides, or it was completely removed to yield a 159 base pair fragment. This second set of alterations resulted in 640 fragments (160 * 4) from each original fragment.

Duplicate fragments in the OP2 design were subsequently identified and removed. These duplicates could have been the result of the removal of a single bp within a region composed of the same nucleotides in Set 3, or they could have been due to overlaps between Set 1 and Set 3.

Connecting barcode to enhancer fragment and plasmid backbone

Paired-end sequence reads were used to connect barcodes to inserted fragments. In all 8 constructed libraries, the barcode was in R2 read and the inserted fragment

(except in the two *_rmBsaI backbones) in R1. The sequence of R2, for example, in pPSup_iGFP_v2 based library begins with: {BBD}AGCTCCTCGCCCTTGCTCACNNBNNBNNBNNBNNBCATGGT, where {BBD} can be either BBD, BD, D, or nothing. The underlined sequences are constant and the NNBx5 is the barcode. In all libraries, R2 starts with the same form, with different constant sequences and different degenerative nucleotides. R1 starts, for example, with the following form in pPSup_iGFP_v2-based library: {BBH}CTTGATATCGAATTCCACTCNNNNNNN.... also here the {BBH} represent up to 3 degenerative bp and the constant sequences is underlined. The NNN... represent the sequence of the inserted fragment. R1 sequences from all libraries have a similar form, with the exception of the two *_rmBsaI-based libraries, where there is no cloned sequence and thus the constant sequence is longer. For pPSint_v2 and pPSint_v2_pTRP1 cloned with OP1+OP2, R1 sequences were of length 210 bp and the read ended with more constant sequences after the up to 160 bp of the synthesized-inserted fragments.

Paired-end reads were filtered to ones that have a maximum of 1 bp mutation in each of the three constant sequences: preceding the inserted fragments in R1, prior to the barcode in R2, or within the 10 bp following the barcode in R2. This means that a read can have 1 bp mutation in all three sequences and still be used. Following the filtering steps the barcode and beginning of the enhancer fragment are extracted. Barcodes that do not fit the VNNx5 pattern are filtered out.

The subsequent processing was carried out independently for OP1+OP2-based libraries and all other libraries. For the latter group containing enhancer fragments, the extracted enhancer fragments were aligned to the 12,000 synthesized oligos using Bowtie (with parameters -v 1 -a –best –strata)[51]. Barcodes associated with more than one enhancer fragment were flagged, and were not used in following analysis. Due to varying sequence coverage and library complexities (particularly between the *_rmBsaI libraries and others), the libraries were downsampled, targeting for 135 appearances of the 50th most frequent barcode.

In the case of OP1+OP2-based libraries, the full 160 bp of the inserted fragment had to be utilized, as OP2 contained sets of all 1 bp mutations derived from specific fragments. Subsequently, paired-reads that passed the initial filtering (i.e., <= 1 bp mutation in constant regions) underwent further filtering to detect the presence of the expected constant sequence ("GAGTAATTGC") after the enhancer, eliminating

less than 5% of reads. All enhancer fragments flanked by constant sequences connected to the same barcode were processed together, to get a mapping of each barcode. To link barcodes to a unique enhancer fragment, the following criteria were applied: (i) The barcode appeared at least in two reads, (ii) The most abundant connected sequence matched one of the synthesized fragments (OP1+OP2) exactly, (iii) None of the other connected sequences with more than 2 bp mismatches to the most abundant sequence were found in the list of synthesized-fragment enhancers (OP1+OP2), (iv) The total number of sequences with up to 2 bp mutations from the most abundant sequence, including the most abundant sequence itself, constituted at least 80% of all sequences connected to the barcode. If a barcode appeared at least twice but couldn't be linked to a single enhancer it was flagged, and was not used in the following analysis.

For the MPRA experiment, groups of libraries were mixed and assayed together. Barcodes from these libraries have to be linked back both to the enhancer and the original library, and if the same barcode appeared in multiple such libraries it cannot be used. To this end, the barcodes to enhancer-fragment dictionaries from each mix of libraries were combined, and barcodes appearing in multiple libraries were marked. Finally, for each of the two mixes of libraries employed in the MPRA experiment, a Tn5-derived DNA sequencing library was generated and sequenced. Barcodes were extracted from the reads based on the following patterns: ACCATG(VNNx5)GTGAGC or GTGATG(VNNx5)GTGAGC. The extracted barcodes were subsequently traced back to their original libraries by searching the corresponding barcode-to-enhancer-fragment dictionaries for each mix, allowing for the inference of actual library-mix ratios.

This procedure produced (1) a dictionary between enhancer X position to barcodes, (2) the normalization weights of how many times each barcode and enhancer appeared in the transformed mix.

MPRA quantification of gene expression

Paired-end reads from the MPRA RNA-Seq libraries were used. The R1 structure from both p35S- and pTRP1-based libraries shared the same structure: {DDHHBDBDHDV}<u>GAACTTGTGGCCGTTTACG</u>. In this sequence, the underlined sections represent constant regions, while the degenerate sequence is a unique molecular identifier (UMI), of length 8 bp up to 11 bp, incorporated during the RT step[52]. In the case of R2, the p35S-based library started with:

{HBB}TTCTAGTATACTAAACCATGVNNVNNVNNVNNVNNGTGAGCAAGG whereas the pTRP1-based library started with: {HHV}TGAGCAATCGAGTGATGVNNVNNVNNVNNVNNGTGAGCAAGG. As described in the previous section, the underlined sequences are constant regions, {HBB} and {HHV} indicate a possible shift of 0-3 bp comprising a random sequence, and VNNx5 represents the barcode sequence. Subsequently, the read-pairs were filtered to include only those exhibiting a maximum of 2-bp mismatch in each of the constant sequences, and having the barcode and UMI in the right lengths. Following this, barcodes and UMIs were extracted from the filtered reads.

The Barcode-UMI pairs were processed in several stages. Firstly, these pairs were collapsed to retain only unique combinations, and the frequency of each unique pair was recorded.

Both the barcode and the UMI contained variable sequences, where specific positions could take on one of three or all four possible nucleotides. This variability allows the estimation of the total sequencing errors in the barcodes or UMIs. With the UMIs used for quantifying expression levels, sequencing errors could potentially lead to inaccurate gene expression estimates.

Given that the MPRA RNA-Seq libraries were sequenced to high effective coverage (sometimes exceeding 20X), it was plausible that a UMI could occasionally be read with a sequencing error. Consequently, the percentage of pairs with a UMI that did not adhere to the variable sequence constraints and a barcode that did was calculated. This calculation was performed at different thresholds of the minimal frequency of barcode-UMI pairs.

The expectation was that setting a higher threshold for the frequency of barcode-UMI pair appearance would also reduce the number of UMIs with sequencing errors. Owing to the degenerate sequence's structure, only one out of three possible errors could be detected for a single base mutation. Therefore, the actual number of UMI sequence errors was assumed to be three times the percentage of detected errors for each frequency threshold.

However, since UMIs with sequences that did not fit the variable sequence could be removed, the real error in estimation was up to double the percentage of detected error sequences. A threshold of less than 0.75% error UMI sequences was set, leading to the potential of up to 1.5% of UMIs being incorrect. It was, however,

expected to be lower due to the RT primers initially containing a UMI that didn't fit the pattern due to errors in the synthesis.

The outcome threshold for appearances from this procedure ranged from 2 to 4 in the various experiments. This approach enabled the minimization of errors while still retaining a significant portion of the data for analysis.

Subsequently, only the barcodes included in the associated mix dictionary were retained. The RNA count for each enhancer X position was determined by the total number of unique barcode-UMI pairs linked to it. To obtain the gene expression level for each construct, this RNA count was normalized by the corresponding normalized weight for the enhancer X position in the mix. Lastly, the total signal for each experiment was normalized to a value of one million, providing a measure parallel to the Transcripts Per Million (TPM) score.

In the majority of the analyses presented in this study, the mean expression of the enhancer, derived from the three or four experimental replicates, is used. For Fig. 2c, the expression of each control enhancer or iUBQ10 was calculated by taking the average gene expression from all its constituent fragments.

Determining overrepresented *k*-mers in downstream enhancers

To uncover overrepresented 6-mers (*k*-mers composed of 6 base pairs) in active downstream enhancers, each species and backbone combination was evaluated separately. Only fragments derived downstream of the TSS were taken into account, and expression levels from downstream positioning were used. For each unique 6-mer (considering reverse complement as well), the enhancers were classified into two groups: those that contained the 6-mer or its complement, and those that did not. A 6-mer was considered in the analysis only if it was present in more than 20 fragments across both groups. The expression levels of the constructs in the MPRA setup were compared between the groups using the Mann-Whitney U test, yielding a p-value. Moreover, the average $\log_2$ expression for each group was determined, and the difference between the groups was assessed. A significance threshold was established at $-\log_{10}(0.05/\#tests)$, where #tests signifies the total number of tests executed for all 6-mers across the eight combinations. This threshold is demonstrated in Fig. 3a and Supplementary Figs. 9,10.

Associations of GATC variation to gene expression in the 1,001G

To detect variations in GATC motifs across the 1,001 Genomes data set, genomic coordinates having a GATC 4-bp sequence within the 1,001 Genomes pseudo-genomes were assembled. The list was filtered down to coordinates within 1 kb of the TSS of genes used in the eQTLs analysis of ref. [1] dataset. For each relevant position, an 8-bp motif-allele was extracted, which includes an extra 2 bp flanking the GATC 4-bp sequence from all pseudo-genomes. Alleles at each site were designated as GATC+ if they aligned with the YVGATCBR motif, and as GATC- if devoid of the GATC sequence. Alleles with a GATC not matching the YVGATCBR motif were excluded. Subsequently, for each identified position, expression values from the two batches from ref. [1] were compared between the GATC+ and GATC- accessions. A statistical analysis was conducted using the Mann-Whitney U test in R for positions with at least 10 elements in both comparison groups. Positions were considered significant if they had a p-value less than 0.05 divided by the total number of tests and showed an average $\log_2$(expression) difference of at least 0.5. Such significant positions were then grouped based on their proximity to the TSS and whether the GATC motif amplified the expression. In total, 111 associations meeting the established criteria were identified. Of the 111 associations identified, distributions for GATC- and GATC+ were: 13 each for -1000 to -500 from the TSS; 17 GATC- and 11 GATC+ for -500 to TSS; 7 GATC- and 18 GATC+ for TSS to +500; and 20 GATC- against 12 GATC+ for +500 to +1000.

Gene ontology enrichment

Gene ontology enrichment (Supplemental Table 7) was performed using DAVID[53], comparing Arabidopsis genes with a GATC motif in the 500 bp downstream of the TSS to all genes.

Evaluation of effect sizes of the GATC motif or other 6-mers

The influence of the GATC motif or 6-mers (as depicted in Fig. 5b) on gene expression and other genomic datasets was determined by analyzing the slope of a linear relationship (defined as the effect size) and the accompanying p-value from the fit. A linear regression, using R’s 'lm' function, was performed to relate the frequency of motif occurrences to the genomic database.

Analysis of gene expression and other genomic measurements from published A. thaliana studies

Meta analysis of different tissue expression in Arabidopsis was done with data from ref. [18], processed as described above, and on a compendium of tissue-specific gene expression data from AtGenExpress, produced with the Affymetrix ATH1 microarray platform, from the Bio-Analytic Resource for Plant Biology (BAR)[17,54]. Due to concerns raised regarding contamination of embryo samples in gene expression datasets[55], the embryo related samples from the latter dataset were replaced with the full compendium of gene expression data collected in a newer dataset[56]. These data also included data from refs. [57,58] which were used in Fig. 5e. In all cases, values of replicates from the same experiment were averaged.

Root expression data at the single-cell level was downloaded from NCBI's GEO database using the accession number GSE152766[59]. Additionally, single-cell data for the vegetative shoot apex was obtained directly via personal correspondence[19]. Both datasets, retaining their initial cell annotations, were processed using Seurat (v4.3.0)[60].

mRNA synthesis rate and mRNA half-lives per gene were used as reported [61].

Data on H3K4me3 and H3K36me3 were from ref. [4]. Using the misha R package, the average signal per gene was calculated and averaged over repeats. RNA polymerase binding information was taken from ref. [62], specifically from the control dataset (NS), which was downloaded from the NCBI's GEO database under the accession number GSE122804[62].

Evolutionary analysis of land plant genomes and transcriptomes

For the evolutionary examination of the GATC motif's impact on genome-wide gene expression, genomes and annotations from the following sources were employed: Arabidopsis - TAIR10[50]; *S. lycopersicum* - ITAG4.0[63]; *O. sativa* - v7[64]; *Z. mays* - B73 NAM-5.0.55[65]; *P. tabuliformis* - v1.0[66]; *C. richardii* - v2.1[67]; *S. moellendorffii* - v1.0[68] annotations from NCBI Annotation Release 100; *M. polymorpha* - v3[69]; *P. patens* - v3.3[70].

Gene expression data were sourced and processed as described below. For *A. thaliana*, raw data were obtained from the SRA database under the accession PRJNA314076[18]. These 138 samples underwent processing through the Nextflow (v22.10.7.5854) tool using the nf-core RNA-Seq pipeline (v3.6) set to default

parameters[71,72]. In the case of tomato, maize, rice, and *P. patens*, processed data were directly downloaded from respective studies[73–76]. For *C. richardii*, the processed dataset was from ref. [77] under the NCBI's GEO database accession GSE212819.

For *P. tabuliformis*, raw RNA-Seq data were accessed from SRA under accession PRJNA173457[66]. Due to the large size of chromosomes (>$2*10^9$ bp), chromosomes were segmented into pseudo-chromosomes no longer than 10^8 bp. Any intersecting features between two consecutive pseudo-chromosomes were discarded. Using GffRead (v0.11.8)[78], a fasta file comprising transcripts was generated from the modified chromosome and annotations. Subsequent quantification of gene expression across the 136 samples employed Salmon (v1.10)[79].

For *S. moellendorffii* and *M. polymorpha*, RNA-Seq datasets were accessed from ref. [80] (SRR1740446 - SRR1740451) and ref. [81] (DRR130762 - DRR130768) in the SRA, respectively. These samples were processed using the nf-core RNA-Seq pipeline similarly to the Arabidopsis samples.

For every gene across each species, a 500 bp sequence downstream of the TSS was extracted, followed by counting the occurrences of the YVGATCBR motif. The effect size and the p-value were calculated using a linear fit as explained above.

Comparing orthologs between Arabidopsis and tomato

Protein sequences from Arabidopsis (TAIR10)[50] and *Solanum lycopersicum* (ITAG4.0)[63] were utilized to conduct orthology analysis using OrthoFinder (version 2.5.4)[15]. Prior to analysis, one variant per gene was selected using the primary_transcript.py tool provided by OrthoFinder. The identification of one-to-one orthologous genes was carried out using the Phylogenetic Hierarchical Orthogroups generated by OrthoFinder, and these orthologs were subsequently employed for further analysis.

Processing of RNA-Seq

RNA-Seq libraries were prepared using the Smart-seq3 protocol[82] , which generates two types of sequence fragments. The first type consists of reads derived from the 5' end of mRNAs, starting with an 11 bp constant sequence (ATTGCGCAATG) followed by a UMI and "GGG". These reads, which represent more than 80% of our library, were used for analysis. The second type, originating from the middle of the mRNAs, lacks a UMI and is thus more susceptible to PCR amplification bias. For UMI processing, UMIs were extracted, and reads (following the "GGG" in R1) that shared identical

UMIs and had up to 2 bp mismatches in read R1 were collapsed using the clumpify.sh script from the BBMap suite[83]. The collapsed reads were then trimmed using Trim Galore with default settings[35]. Gene expression quantification was performed using Salmon (v1.5.2) with the parameters --validateMappings -l A[79]against transcripts from TAIR10 with manual addition of the GFP gene.

For the MPRA libraries of Arabidopsis with MIX1, the same collapsed and trimmed reads were used to assess splicing efficiency.

Assessing splicing efficiency and pTRP1- vs p35S-based total RNA levels

The 5' reads of the Smart-Seq3 library from the full RNA-Seq of the Arabidopsis MPRA experiment with MIX1 were used to assess splicing efficiency. After removing the constant sequence and the UMIs, the reads were filtered to those containing a portion of the sequence between the barcode and the start of the intron (see Extended Data Fig. 2). This was done by taking all 15 bp *k*-mers from this sequence and filtering out all 5' clean reads from the RNA-Seq that had at least one of these *k*-mers, resulting in a total of 20,972 sequences across all four repeats, ranging from 2,735 to 7,092 across the four repeats. When these sequences were further filtered to include sequences from the region upstream of the barcode that differed between the pTRP1 and 35S-based libraries, 90.7% of the reads were uniquely assigned to one of the two libraries. Using the ratio between the number of reads assigned to each library, we estimated the ratio between the backbone without insert of the 35S-based library to be higher than the pTRP1-based by log2 of 3.13, 1.77, 2.3, 2.25 respectively in the four repeats.

The 20,972 5'-end cleaned sequence reads were used to assess splicing efficiency within the MPRA setup. Among these reads, 7,789 R2 sequences contained the sequence "CTTCGCCCTC", which is located just upstream of the splice site and at least 6 bp downstream of this sequence. Of these sequences, 7,405 represented exon-exon junctions and 100 represented exon-intron junctions. Therefore, the estimated splicing efficiency is 98.7% for the four libraries, or 99.39%, 98.91%, 98.55%, and 98.34% for the four repeats, respectively.

References:

1. Kawakatsu, T. *et al.* Epigenomic Diversity in a Global Collection of Arabidopsis thaliana Accessions. *Cell* **166**, 492–505 (2016).
2. Dubin, M. J. *et al.* DNA methylation in Arabidopsis has a genetic basis and shows evidence of local adaptation. *Elife* **4**, e05255 (2015).
3. Clauw, P. *et al.* Leaf Growth Response to Mild Drought: Natural Variation in Arabidopsis Sheds Light on Trait Architecture. *Plant Cell* **28**, 2417–2434 (2016).
4. Liu, Y. *et al.* PCSD: a plant chromatin state database. *Nucleic Acids Res.* **46**, D1157–D1167 (2018).
5. Stroud, H. *et al.* Genome-wide analysis of histone H3.1 and H3.3 variants in Arabidopsis thaliana. *Proc. Natl. Acad. Sci. U. S. A.* **109**, 5370–5375 (2012).
6. Wollmann, H. *et al.* Dynamic deposition of histone variant H3.3 accompanies developmental remodeling of the Arabidopsis transcriptome. *PLoS Genet.* **8**, e1002658 (2012).
7. Chang, K. N. *et al.* Temporal transcriptional response to ethylene gas drives growth hormone cross-regulation in Arabidopsis. *Elife* **2**, e00675 (2013).
8. Vercruyssen, L. *et al.* ANGUSTIFOLIA3 binds to SWI/SNF chromatin remodeling complexes to regulate transcription during Arabidopsis leaf development. *Plant Cell* **26**, 210–229 (2014).
9. Yant, L. *et al.* Orchestration of the floral transition and floral development in Arabidopsis by the bifunctional transcription factor APETALA2. *Plant Cell* **22**, 2156–2170 (2010).
10. 1001 Genomes Consortium. 1,135 Genomes Reveal the Global Pattern of Polymorphism in Arabidopsis thaliana. *Cell* **166**, 481–491 (2016).
11. O'Malley, R. C. *et al.* Cistrome and Epicistrome Features Shape the Regulatory DNA Landscape. *Cell* **165**, 1280–1292 (2016).
12. Reyes, J. C., Muro-Pastor, M. I. & Florencio, F. J. The GATA family of transcription factors

in Arabidopsis and rice. *Plant Physiol.* **134**, 1718–1732 (2004).

13. Schwechheimer, C., Schröder, P. M. & Blaby-Haas, C. E. Plant GATA Factors: Their Biology, Phylogeny, and Phylogenomics. *Annu. Rev. Plant Biol.* **73**, 123–148 (2022).
14. Tu, X. *et al.* Reconstructing the maize leaf regulatory network using ChIP-seq data of 104 transcription factors. *Nat. Commun.* **11**, 5089 (2020).
15. Emms, D. M. & Kelly, S. OrthoFinder: phylogenetic orthology inference for comparative genomics. *Genome Biol.* **20**, 238 (2019).
16. Lin, Z. & Li, W.-H. Evolution of 5' untranslated region length and gene expression reprogramming in yeasts. *Mol. Biol. Evol.* **29**, 81–89 (2012).
17. Schmid, M. *et al.* A gene expression map of Arabidopsis thaliana development. *Nat. Genet.* **37**, 501–506 (2005).
18. Klepikova, A. V., Kasianov, A. S., Gerasimov, E. S., Logacheva, M. D. & Penin, A. A. A high resolution map of the Arabidopsis thaliana developmental transcriptome based on RNA-seq profiling. *Plant J.* **88**, 1058–1070 (2016).
19. Zhang, T.-Q., Chen, Y. & Wang, J.-W. A single-cell analysis of the Arabidopsis vegetative shoot apex. *Dev. Cell* **56**, 1056–1074.e8 (2021).
20. Orkin, S. H. Globin gene regulation and switching: circa 1990. *Cell* **63**, 665–672 (1990).
21. Merika, M. & Orkin, S. H. DNA-binding specificity of GATA family transcription factors. *Mol. Cell. Biol.* **13**, 3999–4010 (1993).
22. Pedone, P. V. *et al.* The N-terminal fingers of chicken GATA-2 and GATA–3 are independent sequence-specific DNA binding domains. *EMBO J.* **16**, 2874–2882 (1997).
23. Newton, A., Mackay, J. & Crossley, M. The N-terminal zinc finger of the erythroid transcription factor GATA-1 binds GATC motifs in DNA. *J. Biol. Chem.* **276**, 35794–35801 (2001).
24. Ko, L. J. & Engel, J. D. DNA-binding specificities of the GATA transcription factor family. *Mol. Cell. Biol.* **13**, 4011–4022 (1993).

25. Shim, Y.-H., Bonner, J. J. & Blumenthal, T. Activity of aC. elegansGATA Transcription Factor, ELT-1, Expressed in Yeast. *J. Mol. Biol.* **253**, 665–676 (1995).
26. Liu, C. *et al.* Molecular Mechanism by Which the GATA Transcription Factor CcNsdD2 Regulates the Developmental Fate of Coprinopsis cinerea under Dark or Light Conditions. *MBio* **13**, e0362621 (2021).
27. Castro-Mondragon, J. A. *et al.* JASPAR 2022: the 9th release of the open-access database of transcription factor binding profiles. *Nucleic Acids Res.* **50**, D165–D173 (2022).
28. Lowry, J. A. & Atchley, W. R. Molecular evolution of the GATA family of transcription factors: conservation within the DNA-binding domain. *J. Mol. Evol.* **50**, 103–115 (2000).
29. Franco-Zorrilla, J. M. *et al.* DNA-binding specificities of plant transcription factors and their potential to define target genes. *Proc. Natl. Acad. Sci. U. S. A.* **111**, 2367–2372 (2014).
30. Weirauch, M. T. *et al.* Determination and inference of eukaryotic transcription factor sequence specificity. *Cell* **158**, 1431–1443 (2014).
31. Xu, Z., Casaretto, J. A., Bi, Y.-M. & Rothstein, S. J. Genome-wide binding analysis of AtGNC and AtCGA1 demonstrates their cross-regulation and common and specific functions. *Plant Direct* **1**, e00016 (2017).
32. Sugimoto, K., Takeda, S. & Hirochika, H. Transcriptional activation mediated by binding of a plant GATA-type zinc finger protein AGP1 to the AG-motif (AGATCCAA) of the wound-inducible Myb gene NtMyb2. *Plant J.* **36**, 550–564 (2003).
33. Liu, Y., Patra, B., Pattanaik, S., Wang, Y. & Yuan, L. GATA and Phytochrome Interacting Factor Transcription Factors Regulate Light-Induced Vindoline Biosynthesis in Catharanthus roseus. *Plant Physiol.* **180**, 1336–1350 (2019).
34. Pisupati, R. *et al.* Verification of Arabidopsis stock collections using SNPmatch, a tool for genotyping high-plexed samples. *Sci Data* **4**, 170184 (2017).

35. Krueger, F. *et al. FelixKrueger/TrimGalore: v0.6.10 - Add Default Decompression Path*. (2023). doi:10.5281/zenodo.7598955.
36. Danecek, P. *et al.* Twelve years of SAMtools and BCFtools. *Gigascience* **10**, (2021).
37. Cheng, C.-Y. *et al.* Araport11: a complete reannotation of the Arabidopsis thaliana reference genome. *Plant J.* **89**, 789–804 (2017).
38. Dobin, A. *et al.* STAR: ultrafast universal RNA-seq aligner. *Bioinformatics* **29**, 15–21 (2013).
39. Purcell, S. PLINK : a toolset for whole-genome association and population-based linkage analysis. *Am. J. Hum. Genet.* **81**, 559–575 (2007).
40. Kang, H. M. *et al.* Efficient control of population structure in model organism association mapping. *Genetics* **178**, 1709–1723 (2008).
41. Voichek, Y. & Weigel, D. Identifying genetic variants underlying phenotypic variation in plants without complete genomes. *Nat. Genet.* **52**, 534–540 (2020).
42. Venables, W. N. & Ripley, B. D. Modern Applied Statistics with S, Springer, New York: ISBN 0-387-95457-0. Preprint at (2002).
43. Zou, Y., Carbonetto, P., Wang, G. & Stephens, M. Fine-mapping from summary data with the 'Sum of Single Effects' model. *PLoS Genet.* **18**, e1010299 (2022).
44. Togninalli, M. *et al.* The AraGWAS Catalog: a curated and standardized Arabidopsis thaliana GWAS catalog. *Nucleic Acids Res.* **46**, D1150–D1156 (2018).
45. Wickham, H. *et al.* Welcome to the tidyverse. *J. Open Source Softw.* **4**, 1686 (2019).
46. Danecek, P. *et al.* The variant call format and VCFtools. *Bioinformatics* **27**, 2156–2158 (2011).
47. Langmead, B. & Salzberg, S. L. Fast gapped-read alignment with Bowtie 2. *Nat. Methods* **9**, 357–359 (2012).
48. Zhang, Y. *et al.* Model-based analysis of ChIP-Seq (MACS). *Genome Biol.* **9**, R137 (2008).
49. Jores, T. *et al.* Identification of Plant Enhancers and Their Constituent Elements by

STARR-seq in Tobacco Leaves. *Plant Cell* **32**, 2120–2131 (2020).

50. Berardini, T. Z. *et al.* The Arabidopsis information resource: Making and mining the 'gold standard' annotated reference plant genome. *Genesis* **53**, 474–485 (2015).
51. Applied Research Applied Research Press. *Ultrafast and Memory-Efficient Alignment of Short DNA Sequences to the Human Genome*. (CreateSpace Independent Publishing Platform, 2015).
52. Kivioja, T. *et al.* Counting absolute numbers of molecules using unique molecular identifiers. *Nat. Methods* **9**, 72–74 (2011).
53. Huang, D. W., Sherman, B. T. & Lempicki, R. A. Systematic and integrative analysis of large gene lists using DAVID bioinformatics resources. *Nat. Protoc.* **4**, 44–57 (2009).
54. Toufighi, K., Brady, S. M., Austin, R., Ly, E. & Provart, N. J. The Botany Array Resource: e-Northerns, Expression Angling, and promoter analyses. *Plant J.* **43**, 153–163 (2005).
55. Schon, M. A. & Nodine, M. D. Widespread Contamination of Arabidopsis Embryo and Endosperm Transcriptome Data Sets. *Plant Cell* **29**, 608–617 (2017).
56. Hofmann, F., Schon, M. A. & Nodine, M. D. The embryonic transcriptome of Arabidopsis thaliana. *Plant Reprod.* **32**, 77–91 (2019).
57. Schneider, A. *et al.* Potential targets of VIVIPAROUS1/ABI3-LIKE1 (VAL1) repression in developing Arabidopsis thaliana embryos. *Plant J.* **85**, 305–319 (2016).
58. Nodine, M. D. & Bartel, D. P. Maternal and paternal genomes contribute equally to the transcriptome of early plant embryos. *Nature* **482**, 94–97 (2012).
59. Shahan, R. *et al.* A single-cell Arabidopsis root atlas reveals developmental trajectories in wild-type and cell identity mutants. *Dev. Cell* **57**, 543–560.e9 (2022).
60. Hao, Y. *et al.* Integrated analysis of multimodal single-cell data. *Cell* **184**, 3573–3587.e29 (2021).
61. Sidaway-Lee, K., Costa, M. J., Rand, D. A., Finkenstadt, B. & Penfield, S. Direct measurement of transcription rates reveals multiple mechanisms for configuration of

the Arabidopsis ambient temperature response. *Genome Biol.* **15**, R45 (2014).

62. Lee, T. A. & Bailey-Serres, J. Integrative Analysis from the Epigenome to Translatome Uncovers Patterns of Dominant Nuclear Regulation during Transient Stress. *Plant Cell* **31**, 2573–2595 (2019).
63. Hosmani, P. S. *et al.* An improved de novo assembly and annotation of the tomato reference genome using single-molecule sequencing, Hi-C proximity ligation and optical maps. *bioRxiv* 767764 (2019) doi:10.1101/767764.
64. Ouyang, S. *et al.* The TIGR Rice Genome Annotation Resource: improvements and new features. *Nucleic Acids Res.* **35**, D883–7 (2007).
65. Hufford, M. B. *et al.* De novo assembly, annotation, and comparative analysis of 26 diverse maize genomes. *Science* **373**, 655–662 (2021).
66. Niu, S. *et al.* The Chinese pine genome and methylome unveil key features of conifer evolution. *Cell* **185**, 204–217.e14 (2022).
67. Marchant, D. B. *et al.* Dynamic genome evolution in a model fern. *Nat Plants* **8**, 1038–1051 (2022).
68. Banks, J. A. *et al.* The Selaginella genome identifies genetic changes associated with the evolution of vascular plants. *Science* **332**, 960–963 (2011).
69. Bowman, J. L. *et al.* Insights into Land Plant Evolution Garnered from the Marchantia polymorpha Genome. *Cell* **171**, 287–304.e15 (2017).
70. Lang, D. *et al.* The Physcomitrella patens chromosome-scale assembly reveals moss genome structure and evolution. *Plant J.* **93**, 515–533 (2018).
71. Ewels, P. A. *et al.* The nf-core framework for community-curated bioinformatics pipelines. *Nat. Biotechnol.* **38**, 276–278 (2020).
72. Di Tommaso, P. *et al.* Nextflow enables reproducible computational workflows. *Nat. Biotechnol.* **35**, 316–319 (2017).
73. Zhang, S. *et al.* Spatiotemporal transcriptome provides insights into early fruit

development of tomato (Solanum lycopersicum). *Sci. Rep.* **6**, 23173 (2016).

74. Stelpflug, S. C. *et al.* An Expanded Maize Gene Expression Atlas based on RNA Sequencing and its Use to Explore Root Development. *Plant Genome* **9**, (2016).
75. Xia, L. *et al.* Rice Expression Database (RED): An integrated RNA-Seq-derived gene expression database for rice. *J. Genet. Genomics* **44**, 235–241 (2017).
76. Perroud, P.-F. *et al.* The Physcomitrella patens gene atlas project: large-scale RNA-seq based expression data. *Plant J.* **95**, 168–182 (2018).
77. Xiao, Y.-L. & Li, G.-S. Differential expression and co-localization of transcription factors during the indirect de novo shoot organogenesis in the fern Ceratopteris richardii. *Research Square* (2023) doi:10.21203/rs.3.rs-2531906/v1.
78. Pertea, G. & Pertea, M. GFF utilities: GffRead and GffCompare. *F1000Res.* **9**, 304 (2020).
79. Patro, R., Duggal, G., Love, M. I., Irizarry, R. A. & Kingsford, C. Salmon provides fast and bias-aware quantification of transcript expression. *Nat. Methods* **14**, 417–419 (2017).
80. Huang, L. & Schiefelbein, J. Conserved Gene Expression Programs in Developing Roots from Diverse Plants. *Plant Cell* **27**, 2119–2132 (2015).
81. Sharma, N., Bhalla, P. L. & Singh, M. B. Transcriptome-wide profiling and expression analysis of transcription factor families in a liverwort, Marchantia polymorpha. *BMC Genomics* **14**, 915 (2013).
82. Hagemann-Jensen, M. *et al.* Single-cell RNA counting at allele and isoform resolution using Smart-seq3. *Nat. Biotechnol.* **38**, 708–714 (2020).
83. Bushnell, B. *BBMap: A Fast, Accurate, Splice-Aware Aligner.* https://www.osti.gov/biblio/1241166 (2014).
