# Supplementary material for: Widespread position-dependent transcriptional regulatory sequences in plants
Source: Nat Genet. 2024 Sep 12;56(10):2238–46. doi: 10.1038/s41588-024-01907-3 (PMC11525189; doi:10.1038/s41588-024-01907-3)
Supplement: Supplementary file 2 — Reporting Summary [file 41588_2024_1907_MOESM2_ESM.pdf]

nature portfolio

Corresponding author(s): Yoav Voicheck & Magnus Norborg

Last updated by author(s): 2024/07/29

Reporting Summary

Nature Portfolio wishes to improve the reproducibility of the work that we publish. This form provides structure for consistency and transparency in reporting. For further information on Nature Portfolio policies, see our Editorial Policies and the Editorial Policy Checklist.

Statistics

For all statistical analyses, confirm that the following items are present in the figure legend, table legend, main text, or Methods section.

n/a	Confirmed	
☐	☒	The exact sample size (*n*) for each experimental group/condition, given as a discrete number and unit of measurement
☐	☒	A statement on whether measurements were taken from distinct samples or whether the same sample was measured repeatedly
☐	☒	The statistical test(s) used AND whether they are one- or two-sided<br>*Only common tests should be described solely by name; describe more complex techniques in the Methods section.*
☒	☐	A description of all covariates tested
☐	☒	A description of any assumptions or corrections, such as tests of normality and adjustment for multiple comparisons
☐	☒	A full description of the statistical parameters including central tendency (e.g. means) or other basic estimates (e.g. regression coefficient) AND variation (e.g. standard deviation) or associated estimates of uncertainty (e.g. confidence intervals)
☐	☒	For null hypothesis testing, the test statistic (e.g. *F*, *t*, *r*) with confidence intervals, effect sizes, degrees of freedom and *P* value noted<br>*Give P values as exact values whenever suitable.*
☒	☐	For Bayesian analysis, information on the choice of priors and Markov chain Monte Carlo settings
☒	☐	For hierarchical and complex designs, identification of the appropriate level for tests and full reporting of outcomes
☐	☒	Estimates of effect sizes (e.g. Cohen's *d*, Pearson's *r*), indicating how they were calculated

Our web collection on statistics for biologists contains articles on many of the points above.

Software and code

Policy information about availability of computer code

Data collection	No software was used for data collection
Data analysis	Trim Galore (v0.6.2), bcftools (v1.16), STAR (v2.7.9), vcftools (v0.1.16), Plink v1.9, GEMMA v0.98.5, MASS R library, SusieR (v0.12.35), misha R package, tidyverse R package, MACS2 v2.2.7.1, Bowtie2, Nextflow v22.10.7.5854, nf-core RNA-Seq pipeline v3.6, Salmon v1.10, Seurat v4.3.0, GffRead v0.11.8, OrthoFinder version 2.5.4, BBMap suite, Trim Galore, Salmon v1.5.2, R's 'lm' function. Custom code: https://zenodo.org/records/12968287

For manuscripts utilizing custom algorithms or software that are central to the research but not yet described in published literature, software must be made available to editors and reviewers. We strongly encourage code deposition in a community repository (e.g. GitHub). See the Nature Portfolio guidelines for submitting code & software for further information.

Data

Policy information about availability of data

All manuscripts must include a data availability statement. This statement should provide the following information, where applicable:

- Accession codes, unique identifiers, or web links for publicly available datasets
- A description of any restrictions on data availability
- For clinical datasets or third party data, please ensure that the statement adheres to our policy

Sequencing data have been deposited in the SRA database with accession number PRJNA1009032. Processed data are available in Supplementary Tables 3-5 and 8.

Published datasets used in this study: SRP074107, Kawakatsu et al. (2016); SRP036643, Dubin et al. (2015); GSE60143, O'Malley et al. (2016); GSE152766, Shahan et al. (2022); GSE122804, Lee & Bailey-Serres (2019); PRJNA314076, Klepikova et al. (2016); GSE212819, Xiao & Li (2023); PRJNA173457, Niu et al. (2022); SRR1740446 - SRR1740451, Huang & Schiefelbein (2015); DRR130762 - DRR130768, Sharma et al. (2013)), 1,001 Genomes Project (VCF file for SNPs, 1001 Genomes Consortium (2016)), TAIR10 (Reference genome and annotations, Berardini et al. (2015), Araport11 (Annotations for gene expression quantification, Cheng et al. (2017)), Plant Chromatin State Database (PCSD) (ChIP-Seq data, Liu et al. (2018)), Bio-Analytic Resource for Plant Biology (BAR) (AtGenExpress tissue-specific gene expression data, Toufighi et al. (2005)).

Research involving human participants, their data, or biological material

Policy information about studies with human participants or human data. See also policy information about sex, gender (identity/presentation), and sexual orientation and race, ethnicity and racism.

Reporting on sex and gender	n/a
Reporting on race, ethnicity, or other socially relevant groupings	n/a
Population characteristics	n/a
Recruitment	n/a
Ethics oversight	n/a

Note that full information on the approval of the study protocol must also be provided in the manuscript.

Field-specific reporting

Please select the one below that is the best fit for your research. If you are not sure, read the appropriate sections before making your selection.

☒ Life sciences ☐ Behavioural & social sciences ☐ Ecological, evolutionary & environmental sciences

For a reference copy of the document with all sections, see nature.com/documents/nr-reporting-summary-flat.pdf

Life sciences study design

All studies must disclose on these points even when the disclosure is negative.

Sample size	MPRA were done in 3 (Maize, Tomato), 4 (Arabidopsis, N. Benthamiana), or 2 (synthesis rate) repeats Overexpression experiments were done in 4 repeats.
Data exclusions	No data was excluded
Replication	Each of the MPRA or O.E experiments were done in 3 or 4 repeats, all replicated experiments were used.
Randomization	Not applicable, as the experiments conducted in this study were performed one at a time.
Blinding	Not applicable, as the experiments conducted in this study were performed one at a time.

Reporting for specific materials, systems and methods

We require information from authors about some types of materials, experimental systems and methods used in many studies. Here, indicate whether each material, system or method listed is relevant to your study. If you are not sure if a list item applies to your research, read the appropriate section before selecting a response.

Materials & experimental systems

n/a	Involved in the study	
☒	☐	Antibodies
☒	☐	Eukaryotic cell lines
☒	☐	Palaeontology and archaeology
☒	☐	Animals and other organisms
☒	☐	Clinical data
☒	☐	Dual use research of concern
☐	☒	Plants

Methods

n/a	Involved in the study	
☒	☐	ChIP-seq
☒	☐	Flow cytometry
☒	☐	MRI-based neuroimaging

Plants

Seed stocks

We used reference accessions. M82 (sp−/sp−), B73, WT N. benthamiana, and Col-0

Novel plant genotypes

n/a

Authentication

n/a
